# Seed size evolution and biogeography of *Plukenetia* (Euphorbiaceae), a pantropical genus with traditionally cultivated oilseed species

**DOI:** 10.1186/s12862-018-1308-9

**Published:** 2019-01-22

**Authors:** Warren M. Cardinal-McTeague, Kenneth J. Wurdack, Erin M. Sigel, Lynn J. Gillespie

**Affiliations:** 1 Department of Biology, University of Ottawa, Gendron Hall, Room 160, 30 Marie Curie, Ottawa, Ontario K1N 6N5 Canada; 2 Research and Collections, Canadian Museum of Nature, PO Box 3443, Station D, Ottawa, Ontario K1P 6P4 Canada; 3 Department of Botany, MRC-166, National Museum of Natural History, Smithsonian Institution, PO Box 37012, Washington, DC 20013-7012 USA; 4 Department of Biology, University of Louisiana at Lafayette, Billeaud Hall, Room 108, 410 E. St. Mary Blvd, Lafayette, LA 70503 USA

**Keywords:** Divergence dating, *KEA1*, Lite Blue Devil, Long-distance dispersal, Plukenetieae, Sacha Inchi, Seed ecology, *TEB*

## Abstract

**Background:**

*Plukenetia* is a small pantropical genus of lianas and vines with variably sized edible oil-rich seeds that presents an ideal system to investigate neotropical and pantropical diversification patterns and seed size evolution. We assessed the biogeography and seed evolution of *Plukenetia* through phylogenetic analyses of a 5069 character molecular dataset comprising five nuclear and two plastid markers for 86 terminals in subtribe Plukenetiinae (representing 20 of ~ 23 *Plukenetia* species). Two nuclear genes, *KEA1* and *TEB*, were used for phylogenetic reconstruction for the first time. Our goals were: (1) produce a robust, time-dependent evolutionary framework for *Plukenetia* using BEAST; (2) reconstruct its biogeographical history with ancestral range estimation in BioGeoBEARS; (3) define seed size categories; (4) identify patterns of seed size evolution using ancestral state estimation; and (5) conduct regression analyses with putative drivers of seed size using the threshold model.

**Results:**

*Plukenetia* was resolved into two major groups, which we refer to as the pinnately- and palmately-veined clades. Our analyses suggest *Plukenetia* originated in the Amazon or Atlantic Forest of Brazil during the Oligocene (28.7 Mya) and migrated/dispersed between those regions and Central America/Mexico throughout the Miocene. Trans-oceanic dispersals explain the pantropical distribution of *Plukenetia*, including from the Amazon to Africa in the Early Miocene (17.4 Mya), followed by Africa to Madagascar and Africa to Southeast Asia in the Late Miocene (9.4 Mya) and Pliocene (4.5 Mya), respectively. We infer a single origin of large seeds in the ancestor of *Plukenetia*. Seed size fits a Brownian motion model of trait evolution and is moderately to strongly associated with plant size, fruit type/dispersal syndrome, and seedling ecology. Biome shifts were not drivers of seed size, although there was a weak association with a transition to fire prone semi-arid savannas.

**Conclusions:**

The major relationships among the species of *Plukenetia* are now well-resolved. Our biogeographical analyses support growing evidence that many pantropical distributions developed by periodic trans-oceanic dispersals throughout the Miocene and Pliocene. Selection on a combination of traits contributed to seed size variation, while movement between forest edge/light gap and canopy niches likely contributed to the seed size extremes in *Plukenetia*.

**Electronic supplementary material:**

The online version of this article (10.1186/s12862-018-1308-9) contains supplementary material, which is available to authorized users.

## Background

**Fig. 1:**
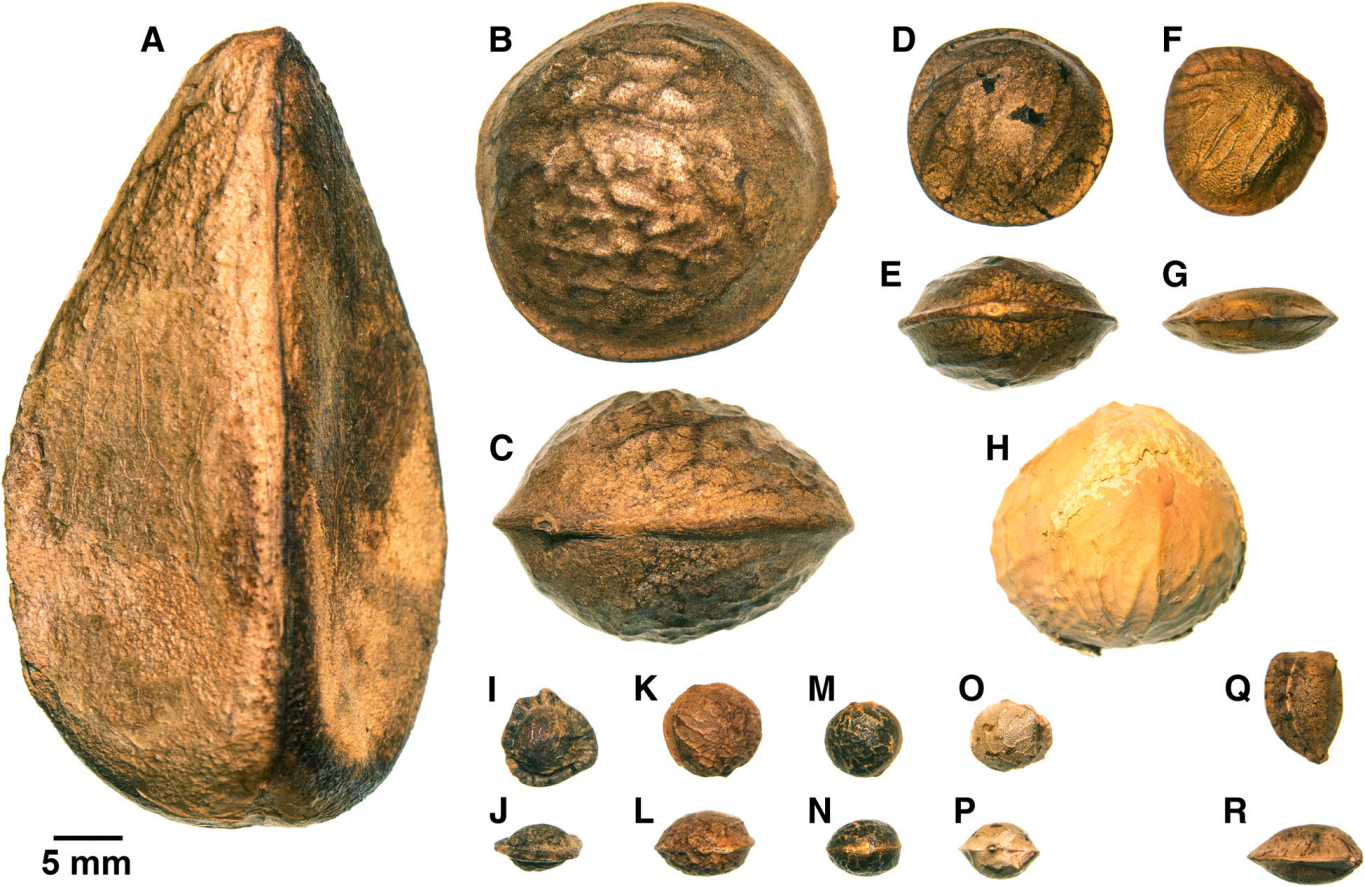
Seed size variation in *Plukenetia* and *Romanoa*. A. *Plukenetia polyadenia* (*van der Werff 16,350*, MO); B–C. *P. huayllabambana* (*Gruhn 84*, MO); D–E. *P. volubilis* (*Nee 35,694*, MO); F–G. *P. stipellata* (*Zambraver 1047*, MO); H. *P. lehmanniana* (*Daniel 4943*, US); I–J. *P. africana* (*Bartsch 1859*, MO); K–L. *P. supraglandulosa* (*Granville 10,783*, US); M–N. *P. loretensis* (*Krukoff 1031*, MO); O–P. *P. verrucosa* (*Barrabé & Crozier 145*, US); Q–R. *Romanoa tamnoides* (*Zardini & Cardozo 44,939*, MO). Dual orientations (face and side) for most species to show flattened lenticular shape. Source: Vouchers in parentheses. Single photograph taken with a Nikon D800

*Plukenetia* L. (Euphorbiaceae subfamily Acalyphoideae) is a small pantropical genus of about 23 species of twining vines and lianas (in rare cases prostrate subshrubs) that represents an ideal system to study tropical biogeography and seed size evolution. Species are found throughout tropical regions of Mexico, Central and South America, Africa, Madagascar, and Southeast Asia [1, 2], making the genus suitable for addressing questions on neotropical diversification patterns and the formation of pantropical distributions. *Plukenetia* seeds exhibit two main qualities that make them desirable for study. First, several species have large edible seeds that are rich in omega-3 and omega-6 polyunsaturated fatty acids and protein content [3–5], making them high-interest crops for both domestic consumption and growing international markets. Second, species of *Plukenetia* exhibit remarkable seed size variation for a genus (Fig. 1), which provides a unique opportunity to investigate the genetic controls and ecological drivers of seed size in a well-defined group of species. Presently, the phylogeny of *Plukenetia*, based on two molecular markers and indel gap-coded data, is missing a significant component of species diversity and requires improved resolution and branch support [6], all of which must be addressed before evolutionary studies can take place on this clade. Here, we develop an improved phylogenetic hypothesis for *Plukenetia* using near-exhaustive taxon sampling across five nuclear and two plastid molecular markers, including two novel low-copy nuclear genes we developed for phylogenetic analysis. This approach allows us to produce a robust time-calibrated phylogeny to examine global patterns of plant biogeography and provide novel insight into the patterns and drivers of seed size evolution among closely related species.

Seed size is an important life-history trait related to mechanisms of dispersal, germination, seedling survival, and the overall reproductive success of plants [7], as well as of economic interest. There have been several macroevolutionary [8–12] and microevolutionary [13–15] seed size studies, but relatively few taxonomic groups with variably sized seeds have been identified and studied on an intermediate level among species or genera (however, see [16–18]). It is unclear if seed size is conserved among most closely related taxa or if it is merely a trait that has been poorly documented at that level. Here, we highlight *Plukenetia* as a tractable, small-sized genus with a growing number of seed-related resources (e.g., oil composition [3–5], germination rate [19–22]) that shows promise for understanding how seed size variation develops among closely related species.

Seed size is influenced by a number of selective pressures including seedling and shade competition, predation, dormancy/persistence, drought tolerance, fire tolerance, and dispersal mechanism [7, 13, 23]. Far less is known about how these selective pressures contribute to patterns of seed size evolution within clades. So far, we know that the seed size of *Hakea* (Proteaceae) is correlated with seed production and post-fire regeneration strategy, but not fruit size, plant height, or the length of time seeds were kept on the plant [17]. By comparison, seed size in *Aesculus* (Sapindaceae) is associated with genome size [18], while in cacti the drivers are not yet known but exclude the amount of light required for germination [16]. Given that closely related species may exhibit phylogenetic constraints on seed size [24], it appears that variation develops through a complex set of interactions between ecological selection and genetic background. Moving forward we should aim to identify the similarities and differences among the numerous contexts in which seed size variation develops.

Seeds of *Plukenetia* are narrowly to broadly lenticular, subglobose, or obovoid and then laterally compressed [1, 2] (Fig. 1) and several traits are potentially correlated with seed size. The most notable trend is the association of smaller seeds in herbaceous vines and slender lianas, and the largest seeds in thick stemmed canopy lianas [1]. In addition, smaller seeded species tend to be associated with light gap and forest edge habitats, whereas the larger seeds of canopy liana species likely germinate and establish under shaded conditions. Fruit type and inferred dispersal mechanism also show potential associations. Smaller seeds are formed in dry, likely explosively dehiscent capsules (as is typical of most euphorbs), which locally disperse high energy seeds that then can be more efficiently dispersed by scatter hoarding rodents [25–27]. In contrast, larger seeds are often associated with green or brown thinly fleshed indehiscent berries, which are likely dispersed by large mammals such as primates that may carry away seeds to eat and occasionally drop a few far from the parent plant [28, 29]. Lastly, while most *Plukenetia* species occur in wet and seasonally moist forests, some species have transitioned into drier habitats, where we find notable seed size reduction associated with fire-prone savanna ecosystems [2].

Within *Plukenetia*, five species have been traditionally cultivated for food and medicine in tropical regions of the Andes (*P. volubilis*, Sacha Inchi or Inca Peanut; *P. carolis-vegae* and *P. huayllabambana*, collectively Mountain Sacha Inchi), the Amazon (*P. polyadenia*, Compadre-de-azeite), and Africa (*P. conophora*, Awusa or African walnut). Despite agricultural interests, the evolutionary origin of large edible oil-rich seeds in *Plukenetia* has not been hypothesized or tested. Large, sometimes oil-rich, seeds have evolved independently in other members of the family [e.g., *Aleurites* J.R. & G.Forst., *Caryodendron* H.Karst., *Hura* L., *Joannesia* Vell., tribe Micrandeae (Mull. Arg.) G.L.Webster, *Omphalea* L.], however most Euphorbiaceae have relatively small seeds. The continental separation of the cultivated species suggests there were at least two independent ancient domestication and/or semi-domestication events in *Plukenetia*. Since large seeded species are present in most taxonomic groupings of *Plukenetia*, we hypothesize that there was a single origin of large seeds in the ancestor of the genus. This hypothesis invokes at least three shifts back to smaller seeds based on the current *Plukenetia* phylogeny [6]. Presently, seed size concepts in *Plukenetia* are informal and oversimplified (e.g., “small versus large”) considering the breadth of variation (Fig. 1). Therefore, measurement-based quantification of seed size variation could help develop a more nuanced interpretation of seed evolution.

The biogeography of *Plukenetia* has not been investigated but could contribute to our understanding of neotropical diversification patterns and the formation of pantropical distributions. There are currently no explicit hypotheses regarding the centre of origin of *Plukenetia*, but it is reasonable to predict that it diversified in South America given that the remaining genera of Plukenetiinae are endemic to that continent. Recent biogeographical analysis of subfamily Acalyphoideae [30] suggests the ancestor of *Plukenetia* was most likely distributed in Central and South America. However, that analysis included only two neotropical species of *Plukenetia* and used biogeographic regions that suited their focus on the Caribbean. Here, we conduct species-level biogeography using more finely parsed South American regions, which may shed light on the impacts that the Andes mountains [31] and open vegetational diagonal [32] had on species movement and diversification patterns. Moreover, biogeographical analysis of *Plukenetia* allows us to test the timing, directionality, and inferred method of movement (e.g., continental migration versus long-distance dispersal) that resulted in its pantropical distributions.

Under its current circumscription, *Plukenetia* is divided into five sections/informal species groups that are found primarily in wet or moist tropical forests (unless otherwise indicated) [1, 2]. They include: (1) sect. *Plukenetia*, containing about eight species from Mexico to Central and South America; (2) a second neotropical species group with about nine species, defined as New World species group “2” by Gillespie [1] (henceforth referred to as NWSG2); (3) sect. *Angostylidium*, containing *P. conophora*, distributed in Africa; (4) sect. *Hedraiostylus*, containing two species (one with an uncommon subshrub growth form) from semi-arid savannas of Africa, and *P. corniculata* from Southeast Asia; and (5) an informal species group from Madagascar [2], containing three species found in dry tropical forest, with two species found exclusively on tsingy limestone. Molecular phylogenetic analysis of Plukenetieae largely supports the morphology-based classification of *Plukenetia*, although species group relationships need improved resolution and support [6].

In this study, we developed a comprehensive phylogeny for *Plukenetia* and closely related genera in subtribe Plukenetiinae to investigate patterns of seed size evolution and biogeography. To produce a well-resolved and well-supported phylogeny, we sampled two plastid and five nuclear DNA markers, including two novel low-copy nuclear genes, one of which was identified using the modified genome/transcriptome data-mining pipeline Lite Blue Devil v0.3. Our goals were to: (1) develop a robust, time-calibrated phylogeny of *Plukenetia* for broad use as an evolutionary framework; (2) reconstruct the biogeographical history of *Plukenetia* and examine hypotheses of neotropical diversification and pantropical disjunct distributions; (3) empirically define seed size categories for *Plukenetia*; (4) perform ancestral seed size estimation to test if there was a single origin of large seeds and identify patterns of seed size variation; and (5) use phylogenetic regression with the threshold model to test putative drivers of seed size evolution.

## Methods

### Taxon sampling for phylogenetic analysis

Our taxon sampling included 86 terminals, 81 of which belong to *Plukenetia* (voucher information and GenBank accessions are provided in Additional file 1: Table S1). We aimed for a comprehensive survey of all known *Plukenetia* species and included multiple accessions of taxa with morphologically diverse species complexes (e.g., *P. brachybotrya*, *P. penninervia*, and *P. volubilis* [1]) and accessions that may represent undescribed species. In total, we sampled 20 of ~ 23 species representing 83% of the total diversity. Species that could not be sampled were either rare and known only from type collections (*P. multiglandulosa*, *P. procumbens*) or material was unavailable (*P. carolis-vegae*). We used five accessions of two closely related taxa in Plukenetiinae (*Haematostemon guianensis* and *Romanoa tamnoides*) as outgroups [6]. The relationship of *Romanoa* as the sister genus of *Plukenetia* is well established with molecular and morphological evidence [1, 6, 33].

### Genome/transcriptome data-mining for novel low-copy nuclear markers

Identification of low-copy nuclear markers largely followed a top-down approach for genome/transcriptome data-mining [34]. This approach operates by conducting a BLAST [35] search of a candidate gene on a designated database of genome/transcriptome sequences followed by alignment and tree building methods to screen for copy number. Potential low-copy nuclear loci were selected from a list of 1083 highly conserved genes identified from the annotated genomes of seven angiosperm species and one moss [36]. We compiled nine previously published genome/transcriptome assemblies for six Euphorbiaceae species: the draft genomes of *Jatropha curcas* [37] and *Ricinus communis* (both coding sequence and gene) [38], five transcriptomes from the 1000 Plants Initiative (i.e., 1KP: *Croton tiglium*, *Euphorbia mesembryanthemifolia* (both juvenile and mature), *Manihot grahamii*, and *R. communis* [39, 40]), and the seed transcriptome of *Plukenetia volubilis* [41] (Additional file 1: Table S2). We used the python script Lite Blue Devil (Additional files 2 and 3; modelled after Blue Devil v0.6 [34]) to detect the longest open reading frames (ORFs) in a series of query sequences (i.e., the list of 1083 highly conserved genes), search for those ORFs within our pool of genome/transcriptome assemblies using BLAST, align the resulting hits using MUSCLE [42], and conduct RAxML [43] BestTree searches on alignments that returned four or more sequences. Lite Blue Devil allows for blastn or blastp searches [35] with specified cut-off values. We used blastn with an e-value threshold of 1e-6. Alignments of potentially low-copy loci were imported into Geneious v8.1.9 (Biomatters, Auckland, New Zealand) and screened for regions of suitable size (400–750 basepairs; bp) with conserved flanking regions to facilitate primer design and amplification. Of the 1083 candidate genes surveyed (see results), AT1G01790, an anticipated potassium (K+) efflux antiporter 1, chloroplastic gene (*KEA1*), demonstrated the highest potential for phylogenetic utility and was carried forward in our study.

### DNA extraction, amplification, and sequencing

Total genomic DNA was extracted from herbarium or silica gel desiccated leaf material using a DNeasy Plant Mini Kit (Qiagen, Valencia, U.S.A.) following the manufacturer’s instructions or with a modified 12 h proteinase K incubation [33, 44]. Marker selection included our newly designed regions and those with previously demonstrated phylogenetic utility in Euphorbiaceae. In total, we sampled seven markers from the nuclear and plastid genomes. The nuclear ribosomal external and internal transcribed spacers (ETS, ITS) and partial *matK* regions have been used within Plukenetieae [6, 45], while full or partial *matK* and *ndhF* regions have been broadly applied across Euphorbiaceae and Acalyphoideae [30, 46–48]. Low-copy nuclear markers *KEA1* introns 11 and 17 (designed here) and *TEB* exon 17 (designed earlier by KJW) have not been previously used for phylogenetic analyses. *TEB* (AT4G32700) was originally screened but found unsatisfactory (i.e., erratic gene recovery) in preliminary work on broad Malpighiales phylogenetics (see [49]). We also redesigned Euphorbiaceae-specific primers for ETS and *matK*. The ETS primers were designed from ribosomal DNA assemblies from whole genome libraries of *Haematostemon guianensis* run on the Ion Torrent platform (Thermo Fisher Scientific) (KJW, unpublished data). The *matK* primers were designed by identifying conserved regions on an alignment with representatives of *Acalypha*, *Bernardia*, *Caryodendron*, *Ricinus*, and genera from tribe Plukenetieae. Additional file 1: Table S3 outlines the primers and parameters used for amplification and sequencing [50–55]. Additional file 4: Figure S1 illustrates new or redesigned markers and their relative primer positions. PCR products were treated with an exonuclease I (Exo) and shrimp alkaline phosphatase (SAP) procedure (MJS Biolynx, Brockville, Canada) or with ExoSAP-IT (Fisher Scientific, Fair Lawn, U.S.A.), followed by Sanger sequencing with BigDye Terminator v3.1 chemistry (Applied Biosystems, Foster City, U.S.A.). Sequencing reaction products were cleaned with a sodium acetate/ethanol precipitation or Sephadex G-50 (GE Healthcare Bio-Sciences, Pittsburg, U.S.A.) and run on an ABI 3130xl Genetic Analyzer (Applied Biosystems) at the Laboratory of Molecular Biodiversity (Canadian Museum of Nature) or at the Laboratories of Analytical Biology (Smithsonian Institution, National Museum of Natural History). All consensus sequences were assembled and edited using Geneious.

### Sequence alignment and model selection

Sequences were aligned using the auto-select algorithm of MAFFT v7.017 [56] in Geneious, then manually adjusted using a similarity criterion [57]. Optimal models of molecular evolution were selected for each marker by ranking the maximum likelihood (ML) scores of 24 nucleotide substitution models under PhyML [58] BEST tree searches using the Akaike information criterion (AIC; [59]) implemented in jModelTest v2.1.10 [60]. The GTR + I + G model was selected for ITS and *ndhF*, GTR + G for *KEA1* intron 11 and *matK*, HKY + G for ETS, and HKY + I for *KEA1* intron 17 and *TEB* exon 17.

### Phylogenetic analyses

Prior to conducting phylogenetic analyses on combined datasets, we tested for well-supported incongruence between individual markers using ML bootstrap analyses [61] conducted in Garli v2.01 on XSEDE [62] through the CIPRES Scientific Gateway v3.3 [63] (all other analyses were run with desktop programs). For each marker we generated 500 bootstrap replicates, implementing two independent runs starting from random trees, terminating after 20,000 generations without topological improvement, and estimating the values of each model of molecular evolution. A 50% majority-rule consensus tree was made from the optimal trees recovered from each replicate using the Consensus Tree Builder in Geneious. Individual marker bootstrap values were mapped onto the optimal ML tree recovered under the same search conditions. Topologies from individual analyses were evaluated by pairwise comparisons, searching for well-supported conflicting relationships (interpreted as ≥85 ML bootstrap percentage; MLBP).

We implemented parsimony and Bayesian approaches for the analysis of concatenated datasets. Parsimony analyses were executed in PAUP* v4.0b10 [64] with characters treated as unordered and equally weighted [65]. Branch support was evaluated using 1000 bootstrap replicates, each with 10 random-addition replicates, applying tree-bisection-reconnection (TBR) swapping, saving multiple shortest trees each step (Multrees), with each replicate limited to the first 100 shortest trees. Bayesian Markov chain Monte Carlo (MCMC) analyses were conducted in MrBayes v3.2.2 [66] on concatenated datasets partitioned by marker and with independently estimated models. Two independent runs of four-chained searches were performed for three million generations, sampling every 1000 generations, with the remaining search parameters at their default settings. Independent runs were considered converged when the standard deviation of split frequencies were < 0.005, potential scale reduction factors (PSRF) were near 1.0, and effective sample size (ESS) values of each parameter were > 200 (determined using Tracer v1.6 [67]). The first 25% of each run (750 trees) was discarded as burn-in prior to summarizing a maximum clade credibility tree and calculating posterior probabilities (PP).

### Divergence date estimation

Molecular dating analyses were assessed under a relaxed molecular clock using Bayesian methods in BEAST v1.8.0 [68]. XML files were prepared in BEAUti v1.8.0 as a partitioned seven-marker dataset with independently estimated models of nucleotide evolution. We used two uncorrelated lognormal (UCLN) relaxed clock models separated into nuclear and plastid divisions, independently estimating rates of molecular evolution and rate variation parameters. The UCLN mean rate priors were set as a uniform distribution (0 to 1.0e100) with an initial value of 1.0. A single tree was modeled starting from a random tree and using the Yule process of speciation [69]. Since there are no known *Plukenetia* fossils, we used two secondary ‘time to most recent common ancestor’ (TMRCA) calibration priors based on molecular dating estimates of subfamily Acalyphoideae that used three fossil calibrations [30]. We used normal-distribution priors to constrain the root of the tree and crown of Plukenetiinae (which are the same node) to 36.43 Mya (SD = 4.0) and the crown of *Plukenetia* to 15.21 Mya (SD = 4.0). We initiated three independent MCMC runs for 21 million generations, sampling every 10,000 generations. Runs were assessed for convergence and ESS > 200 using Tracer. When convergence and ESS thresholds were met, runs were combined after excluding the first million generations as burn-in using LogCombiner v1.8.0. A maximum clade credibility tree with mean ages was summarized in TreeAnnotator v1.8.0 with a PP limit of 0.95.

### Ancestral range estimation

We evaluated potential historical distributions patterns using ancestral range estimation (ARE). We limited biogeographical analyses to species lineages by pruning replicate species tips off the maximum clade credibility BEAST tree using the drop.tip function of phytools [70] in R v3.3.2 [71]. Species distributions were gathered from literature [1, 2, 72–75] and online [76, 77] resources. We categorized six areas of distribution: (1) Mexico, Central America, and Northwestern South America (north and west of the Andes); (2) the Amazon biome, including the Guiana shield; (3) the Atlantic Forest biome (Mata Atlântica, Brazil); (4) tropical wet and semi-arid Africa; (5) Madagascar; and (6) Southeast Asia (Additional file 1: Table S4). Our neotropical areas were adapted from Morrone [78] to subdivide the South American floral kingdom, whereas our paleotropical areas were largely defined by the African and Indo-Pacific floral kingdoms [79], except with the recognition of Madagascar as a distinct region from Africa.

We conducted ARE on the modified BEAST chronogram using the maximum-likelihood approach in BioGeoBEARS [80, 81] implemented in R. Our analyses used the dispersal-extinction-cladogenesis (DEC) model from Lagrange [82] in addition to the founder-event parameter (+J) developed in BioGeoBEARS. The +J parameter allows for “jump speciation”, in which cladogenic dispersals can occur outside of the parental area. For recent and ongoing discussion over criticisms of the DEC + J model, see Ree and Sanmartín [83]. Dispersal probabilities between pairs of areas were specified by distance for a single timeslice (Additional file 1: Table S5) following similar analyses [84–87]. To facilitate clear patterns, inferred ancestral ranges were allowed to occupy up to two areas. We conducted two independent runs with the DEC and DEC + J models and used a likelihood-ratio test to determine the model of best fit.

### Defining seed size categories

To empirically define seed size categories, we compiled seed dimension measurements (length, width, and thickness) for all species of *Plukenetia* using literature reports [1, 2, 72–75] and/or mature seeds of (usually) dehisced fruits from dried herbarium specimens. The dimensions were converted into estimated volumes based on the formula for an ellipsoid (*v* = 4/3 π abc).

We used clustering analysis as an objective approach to identify seed size categories using PAST v3.14 [88]. We clustered individual seeds based on their log_10_ transformed dimension measurements using Euclidean distance and the unweighted pair-group method using arithmetic averages (UPGMA). Clusters were defined by a distance of ~ 0.35. We visualized the resulting clusters using a three-dimensional principal components analysis (PCA).

### Models of continuous trait evolution

We examined patterns of seed size evolution by fitting the modified BEAST chronogram with the mean log_10_(estimated seed volumes) for each species under three models of trait evolution: (1) Brownian motion; (2) Ornstein-Uhlenbeck (OU); and (3) Early Burst (EB). *Plukenetia huayllabambana* was included in seed evolution analyses by using a placeholder that shared the same phylogenetic position within the nuclear tree. We fit the data to each model using the fitContinuous function in the R package geiger [89] and then ranked the best model using ΔAIC and Akaike weights (*w*_*i*_; [90]). We assessed the phylogenetic signal of our data with Blomberg’s *K* [91] and Pagel’s λ [92] using the phylosig function of phytools.

### Ancestral state estimation

We performed maximum likelihood ancestral state estimation of log_10_(seed size volume) as a continuous character on the modified BEAST chronogram using the contMap function in phytools. The contMap function uses ML to estimate the ancestral state of internal nodes under Brownian motion, then interpolates the states along the edges of each branch with Felsenstein’s eq. (3) [93, 94]. We also visualized seed size evolution through time by plotting the log_10_(estimated seed volume) on the modified BEAST chronogram using the phenogram function in phytools.

### Phylogenetic regression analyses

We used phylogenetic regression to test the correlation between seed size and five putative drivers of seed evolution: plant size, fruit type/dispersal mechanism, seedling ecology, fire tolerance, and biome type. Due to limited quantitative data, we simplified the complexity of each trait into a binary character (Additional file 1: Table S6). Plant size was divided into species with slender (0) versus robust to thick (1) stems; fruit type/dispersal mechanism into dry and dehiscent (0) versus fleshy and indehiscent (1); seedling ecology by light gap and forest edge establishment (0) versus a shade avoidance canopy liana strategy (1); fire tolerance by species that are non-fire adapted (0) and fire adapted (1); and biome type by species that occupy wet dominate biomes (0) versus biomes with a significant dry component (1).

Correlation analyses were conducted with the threshold model [95] using the threshBayes function in phytools. The threshold model includes an unobserved quantitative character called the liability, where a trait is determined by whether the liability passes an arbitrary threshold. This method uses BI to measure the covariation of trait liabilities, allowing for the comparison of both continuous and discrete binary data. We ran MCMC analyses for 3 million generations sampling every 1000th step and applied a 20% burnin prior to summarizing posterior distribution values for the correlation coefficient (*r*). We assessed the ESS of the coefficient using the effectiveSize function in the R package coda [96].

## Results

### Genome/transcriptome data-mining results

Of the 1083 candidate conserved genes queried through Lite Blue Devil, 222 were returned and six were identified as potentially appropriate low-copy markers: AT1G01790, AT1G06820, AT1G08520, AT1G13820, AT2G26680, AT3G11830, AT3G54670. We designed 12 primer pairs across those candidate genes using *Plukenetia* and *Ricinus* database sequences as templates. After preliminary tests of amplification success, low-copy status (i.e., single band and few polymorphisms), and nucleotide variation, AT1G01790 (*KEA1*) was identified as most suitable for use in phylogenetic reconstructions.

### Phylogenetic relationships

**Fig. 2:**
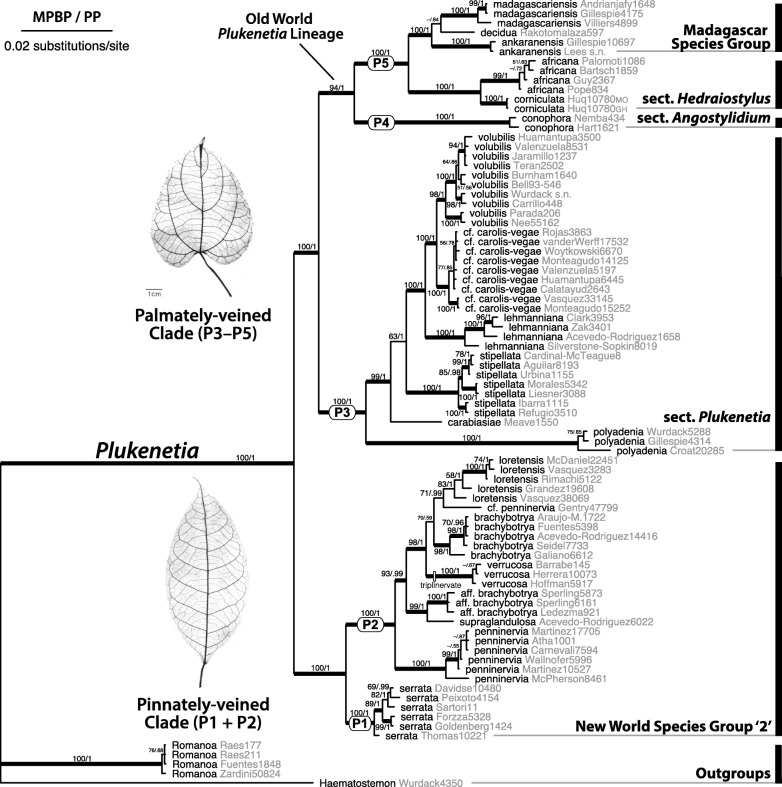
Bayesian maximum clade credibility tree based on the combined seven marker (cpDNA and nDNA), 83 accession dataset for *Plukenetia* and Plukenetiinae outgroups. Maximum parsimony bootstrap percentage (MPBP) and Bayesian posterior probabilities (PP) support values > 50% are indicated on each branch. Branches in bold indicate strong support (≥ 85 MPBP and ≥ 0.95 PP). Subclade numbering system (P1–P5) follows Cardinal-McTeague and Gillespie [6]. Inset, leaf clearings demonstrating pinnately-veined (*P. supraglandulosa*, *Granville 3626*, CAY) and palmately-veined (*P. stipellata*, *Gillespie 413*, US) leaf architecture

Table 1 presents dataset characteristics for each molecular marker. Comparisons of individual markers’ bootstrap analyses did not recover any well supported topological conflicts (Additional file 4: Figure S2). As such, further analyses were conducted on three concatenated datasets: (i) combined plastid (cpDNA; *matK*, *ndhF*) including 74 accessions; (ii) combined nuclear (nDNA; ETS, ITS, *KEA1* introns 11 and 17, *TEB* exon 17) including 86 accessions; and (iii) total combined (all seven markers, plastid + nuclear) including 83 accessions. The total combined matrix had an aligned length of 5069 characters, 2660 of nDNA and 2409 of cpDNA (Table 1). Comparison of cpDNA and nDNA topologies revealed two instances of well supported conflicting topologies (Additional file 4: Figure S3), resulting in the removal of all *Plukenetia huayllabambana* accessions (*Téllez 4*, *Quipuscoa 381*) and one accession of *P. loretensis* (*Solomon 7972*) from the total combined dataset. Aside from these shallow incongruences, topologies were consistent across analyses and datasets, with greatly increased node support in total combined analyses (Fig. 2). Hereafter, the results and discussion focus on the total combined dataset (Fig. 2) and refer to the *Plukenetia* subclade naming system established by Cardinal-McTeague and Gillespie [6].

**Table 1:** Molecular dataset characteristics

Dataset	n ITS	n ETS	n *KEA1* intron 11	n *KEA1* intron 17	n *TEB* exon 17
No. terminals	85	84	73	57	45
Aligned length	803	468	435	396	558
Variable characters	304	241	150	88	141
Parsimony informative characters (%)	266 (33%)	199 (43%)	121 (28%)	68 (17%)	90 (16%)
Nucleotide model	GTR + I + G	HKY + G	GTR + G	HKY + I	HKY + I
Dataset	cp *matK*	cp *ndhF*	Combined plastid	Combined nuclear	Total combined
No. terminals	70	70	74	86	83
Aligned length	1654	755	2409	2660	5069
Variable characters	175	128	303	924	1227
Parsimony informative characters (%)	115 (7%)	94 (13%)	209 (9%)	744 (28%)	953 (19%)
Nucleotide model	GTR + G	GTR + I + G	–	–	–

General relationships across subtribe Plukenetiinae are strongly supported (interpreted as maximum parsimony bootstrap percentage [MPBP] ≥ 85, PP ≥ 0.95, indicated by bold branches) with only three internal nodes with low support (Fig. 2). *Romanoa*, *Plukenetia*, and each of the *Plukenetia* subclades (P1–P5) are monophyletic with strong support (MPBP = 100, PP = 1.0). *Plukenetia* is resolved into two main clades with strong support (MPBP = 100, PP = 1.0) that we formally name (i) the pinnately-veined clade (P1 + P2), containing NWSG2, and (ii) the palmately-veined clade (P3–P5), including sect. *Plukenetia* (P3) + the Old World *Plukenetia* lineage (P4 + P5). The latter comprises sect. *Angostylidium* (P4) and sect. *Hedraiostylus* + the Madagascar species group (P5).

Species relationships are well-resolved and a majority are strongly supported (Fig. 2). Within the pinnately-veined clade, subclade P1 (*P. serrata*) and subclade P2 (the remaining members of NSWG2) form a strongly supported sister group (MPBP = 100, PP = 1.0). The earliest diverging lineage of subclade P2, *P. penninervia*, is sister to two strongly supported clades composed of: (i) *P.* aff. *Brachybotrya* + *P. supraglandulosa*; and (ii) a functional polytomy containing *P. brachybotrya*, *P. verrucosa*, and a weakly supported clade (MPBP = 70, PP = 0.59) of *P.* cf. *penninervia* + *P. loretensis*. Within the palmately-veined clade, subclade P3 (sect. *Plukenetia*) is sister to P4 + P5 (the Old World *Plukenetia* lineage) with strong support (MPBP = 94 or 100, PP = 1.0). Sect. *Plukenetia* (P3) is monophyletic (MPBP = 100, PP = 1.0) and resolved into a successive grade of *P. polyadenia*, *P. carabiasiae*, *P. stipellata*, and *P. lehmanniana*, of which the latter is sister to *P.* cf. *carolis-vegae* + *P. volubilis*. The Old World *Plukenetia* lineage (P4 + P5) is monophyletic with strong support (MPBP = 94, PP = 1.0), with sect. *Angostylidium* (P4; *P. conophora*) sister to sect. *Hedraiostylus* (P5; *P. africana* + *P. corniculata*) + the Madagascar species group (P5; a polytomy of *P. ankaranensis*, *P. decidua*, and *P. madagascariensis*), all with strong support (MPBP = 100, PP = 1.0).

### Divergence dating

**Fig. 3:**
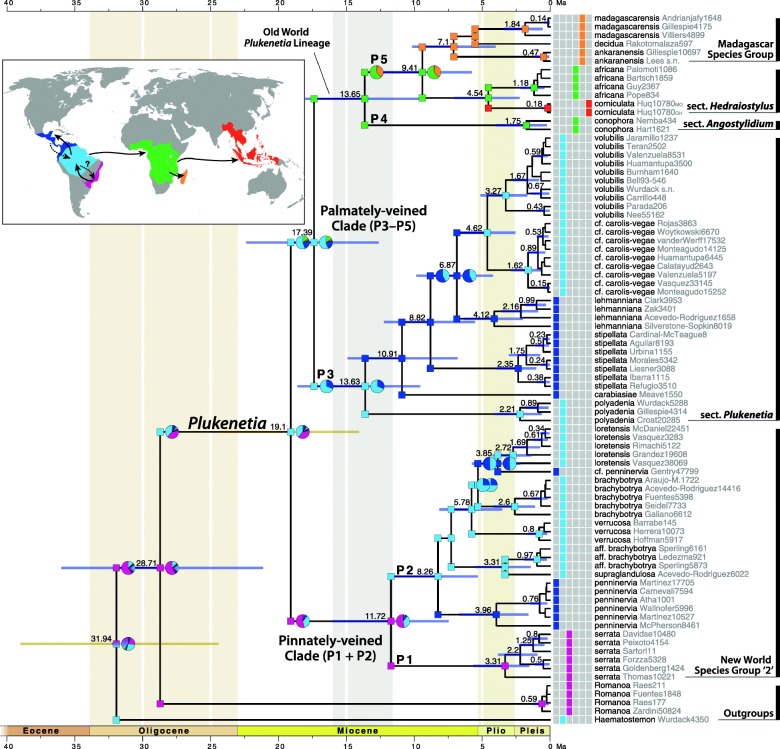
Ancestral range estimation on the *Plukenetia* BEAST chronogram using BioGeoBEARS (DEC + J model). Areas of tip species are shown left of taxa names, color-coded for the six biogeographical areas depicted on the map inset. Boxes at each node and corner are color-coded for the area (or combined area, up to two allowed) with the highest maximum likelihood probability. Pie charts indicate the probability of each area and are included when there is < 75% confidence for a single area. Numbers at each node indicate mean age estimates, blue bars the 95% highest posterior density (HPD) confidence interval, and yellow bars the 95% HPD of calibrated nodes estimated under constraint. Arrows on the map indicate the general direction of range movement in *Plukenetia*; dashed lines indicate area reversals

The BEAST chronogram is presented with mean age estimates and 95% highest posterior density (HPD) confidence bars for nodes with ≥0.95 PP (Fig. 3). A simplified BEAST chronogram is illustrated in Additional file 4: Figure S4. The crown of Plukenetiinae is estimated (under constraint) to have diverged in the Oligocene (31.94 Mya, HPD = 39.01–24.42) with *Romanoa* and *Plukenetia* diverging at 28.71 Mya (HPD = 36.0–21.12). The crown of *Plukenetia* (estimated under constraint) diverged in the Early Miocene (19.1 Mya, HPD = 24.11–14.07) with sect. *Plukenetia* (P3) and the Old World *Plukenetia* lineage (P4 + P5) diverging at 17.39 Mya (HPD = 22.38–12.62). Species of *Plukenetia* diverged continuously from the Middle Miocene (16.0 to 11.6 Mya) into the Pliocene (5.3 to 2.6 Mya).

### Reconstructing biogeographical history

ARE analyses using the DEC and DEC + J models recovered similar overall patterns but with less resolved deep-node estimates under the DEC model. A likelihood-ratio test between the two models revealed inclusion of the +J “jump speciation” parameter significantly improved likelihood scores (DEC lnL = − 46.67, DEC + J lnL = − 27.26, χ^2^(1) = 38.83, *p* = 4.6e-10). As such, only the DEC + J results are presented (see Additional file 4: Figure S5 for complete DEC + J and DEC outputs, and [83] for recent criticisms of the DEC + J model). The resulting parameters of the DEC + J model included: anagenetic dispersal rate *d* = 1e-5, extinction rate *e* = 1e-5, and cladogenetic dispersal rate *j* = 0.3997152. ARE on the *Plukenetia* BEAST chronogram using BioGeoBEARS is shown in Fig. 3, illustrating the area or combined areas with highest probability for each node and corner. Nodes/corners with low area probabilities (< 0.75 PP) include pie charts depicting the proportion of probable areas (Fig. 3).

Biogeographical hypotheses inferred from ARE are based on several nodes with high probability, although the proportion of probable areas for the common ancestor of *Plukenetia* is split between two regions (Fig. 3). Biogeographical history at the crown of Plukenetiinae has low resolution but involved the Amazon and Atlantic Forest regions, either separately or as a combined area. The stem lineages of each genus diverged during the Oligocene (33.9 to 23.0 Mya) after becoming isolated in the Amazon (*Haematostemon*) or Atlantic Forest (common ancestor of *Plukenetia* + *Romanoa*) regions. The crown of *Plukenetia* diverged rapidly in the Early Miocene (23.0 to 16.0 Mya) most probably in the Amazon or Atlantic Forest.

ARE within *Plukenetia* revealed frequent migrations/dispersals within the pinnately- (P1 + P2) and palmately-veined (P3–P5) clades. Two biogeographical histories are highly likely in the ancestor of *Plukenetia*. In one scenario, *Plukenetia* originated after its ancestor migrated from the Atlantic Forest into the Amazon during the Oligocene, after which the pinnately-veined clade (P1 + P2) diverged and returned to the Atlantic Forest in the Early Miocene. In the other scenario, *Plukenetia* originated in the Atlantic Forest followed by the ancestor of the palmately-veined clade (P3–P5) migrating into the Amazon in the Early Miocene. Within the pinnately-veined clade (P1 + P2) the ancestor of subclade P1 remained in the Atlantic Forest, while the ancestor of subclade P2 migrated/dispersed to the Amazon by the end of the Middle Miocene followed by two independent migrations/dispersals to Central and NW South America in the Late Miocene (11.6 to 5.3 Mya) and Pliocene. Within sect. *Plukenetia* (P3) an early diverging lineage migrated/dispersed into Central and NW South America in the Middle Miocene and subsequently diversified. The common ancestor of *P.* cf. *carolis-vegae* + *P. volubilis* migrated back into the Amazon during the Pliocene. The ancestor of the Old World *Plukenetia* lineage (P4 + P5) most likely underwent a long-distance trans-Atlantic Ocean dispersal from the Amazon to tropical Africa during the Early Miocene. Within subclade P5 an early diverging lineage underwent a short-distance dispersal into Madagascar during the Late Miocene, with a later diverging lineage undergoing a long-distance trans-Indian Ocean dispersal to Southeast Asia in the Pliocene.

### Seed measurements and seed-size categories

In total, we sampled 122 vouchers of *Plukenetia*, *Romanoa*, and *Haematostemon* and produced a dataset of 212 individual seed measurements (Additional file 5), which are summarized for each species in Table 2.

**Table 2:** Summary of seed measurements for *Plukenetia*, *Romanoa*, and *Haematostemon* (min–max [mean]). Size categories: (S) small, 25–100 mm^3^; (M) medium, 100–500 mm^3^; (L) large, 500–3000 mm^3^; (XL) extra-large, 3000–13,000 mm^3^; (Max) maximum, 26,000–38,000 mm^3^. † = measurements based solely on the species description; n/a = seeds not known

Taxon	No. seeds	Length (mm)	Width (mm)	Thickness (mm)	Estimated volume (mm^3^)	Size category
*Haematostemon guianensis* Sandw.	1	4	4	4.5	38	S
*Plukenetia africana* Sond.	18	5.5–8 (6.6)	5–7.5 (6.5)	2.5–4 (3.4)	47–126 (79)	S–M
*P. ankaranensis* L.J.Gillespie	6	15–18 (16.5)	16–17 (16.4)	14–17 (15.4)	1929–2623 (2187)	L
*P. brachybotrya* Müll.Arg.	5	5–6 (5.7)	4–5.5 (4.9)	4–5 (4.5)	42–79 (67)	S
*P*. aff. *Brachybotrya*	4	5.5–5.6 (5.6)	4–4.9 (4.6)	3.3–4.1 (3.7)	37–59 (49)	S
*P. carabiasiae* J.Jiménez Ram.	†	24–27	21–27	14–16	3695–6107	XL
*P. carolis-vegae* Bussmann, Paniagua & C.Téllez	1	27	25	20	7069	XL
*P.* cf. *carolis-vegae*	2	19–20	17–18.6	13–15.8	2221–2981 (2601)	L
*P. conophora* Müll.Arg.	3	25–29 (27)	25–27 (26.3)	25–28 (26)	8181–10,688 (9706)	XL
*P. corniculata* Sm.	27	8–10.5 (9.3)	6–8 (7.3)	4–8 (5.5)	123–272 (197)	M
*P. decidua* L.J.Gillespie	2	13.1	11.1–11.2 (11.2)	11.2–11.8 (11.5)	853–907 (880)	L
*P. huayllabambana* Bussmann, C.Téllez & A.Glenn	2	25–28 (26.5)	22–27 (24.5)	17–20 (18.5)	4896–7917 (6627)	XL
*P. lehmanniana* (Pax & K.Hoffm.) Huft & L.J.Gillespie	3	28–34.3 (30.8)	20–24 (21.7)	24–30 (26)	7037–12,919 (9289)	XL
*P. loretensis* Ule	12	4.5–6 (5.1)	4–5.1 (4.5)	3–5 (4)	28–79 (48)	S
*P. madagascariensis* Leandri	n/a	n/a	n/a	n/a	n/a	n/a
*P. multiglandulosa* Jabl.	n/a	n/a	n/a	n/a	n/a	n/a
*P. penninervia* Müll.Arg.	8	5–6 (5.3)	5–6 (5.6)	4–5 (4.5)	60–79 (70.6)	S
*P.* cf. *penninervia*	2	7	6.3–6.5	5–5.2	115–124 (120)	M
*P. polyadenia* Müll.Arg.	19	49–56 (51.3)	33–37 (34.8)	30–36 (32.7)	26,177–37,322 (30,684)	Max
*P. procumbens* Prain	n/a	n/a	n/a	n/a	n/a	n/a
*P. serrata* (Vell.) L.J.Gillespie	2	15–15.5 (15.3)	15.5–16 (15.8)	15–16 (15.5)	1887–2011 (1949)	L
*P. stipellata* L.J.Gillespie	47	10–14 (11.8)	8–13.5 (10.2)	2.5–6 (4.7)	144–484 (301)	M
*P. supraglandulosa* L.J.Gillespie	3	7–7.3 (7.2)	6.8–7.1 (6.9)	4.6–4.8 (4.7)	117–125 (122)	M
*P. verrucosa* Sm.	10	5.3–6 (5.9)	3.7–5.5 (5.1)	3.5–5 (4)	55–69 (62)	S
*P. volubilis* L.	14	13–22 (17)	11.8–18 (15.2)	5–9 (7.2)	480–1659 (997)	(M) L
*Romanoa tamnoides* (A.Juss.) Radcl.-Sm.	21	6.3–8 (7.3)	5–6 (5.6)	3–4.2 (3.9)	63–101 (83)	S

Clustering analysis identified five size-based groups in *Plukenetia* (Additional file 4: Figure S6) that can be classified by estimated seed volume: (S) small, 25–100 mm^3^; (M) medium, 100–500 mm^3^; (L) large, 500–3000 mm^3^; (XL) extra-large 3000–13,000 mm^3^; and (Max) maximum 26,000–38,000 mm^3^. PCA analysis revealed the first component (PC1) accounted for 95% of the observed variance, suggesting limited overlap on the x-axis is a good measure of discreteness (Additional file 4: Figure S7). We note that other seed size categories could be defined based on clustering or PCA analysis, but these volume-based categories provide intuitive boundaries based on the seed size variation of *Plukenetia*. The seed size variance of most species could be attributed to a single category, with the exception of *P. africana* (evenly split between M and S) and *P. volubilis* (mostly L but rarely M).

### Patterns of seed size evolution

Seed size exhibited strong phylogenetic signal under Blomberg’s *K* (1.0173; *p* = 0.001) and Pagel’s λ (0.9999; *p* = 0.0004), with statistical values strongly suggestive of the Brownian motion model of trait evolution. AIC also identified Brownian motion as the best model of evolution (*w*_*i*_ = 0.66), rather than OU or EB (*w*_*i*_ = 0.17, each) (Additional file 1: Table S7).

**Fig. 4:**
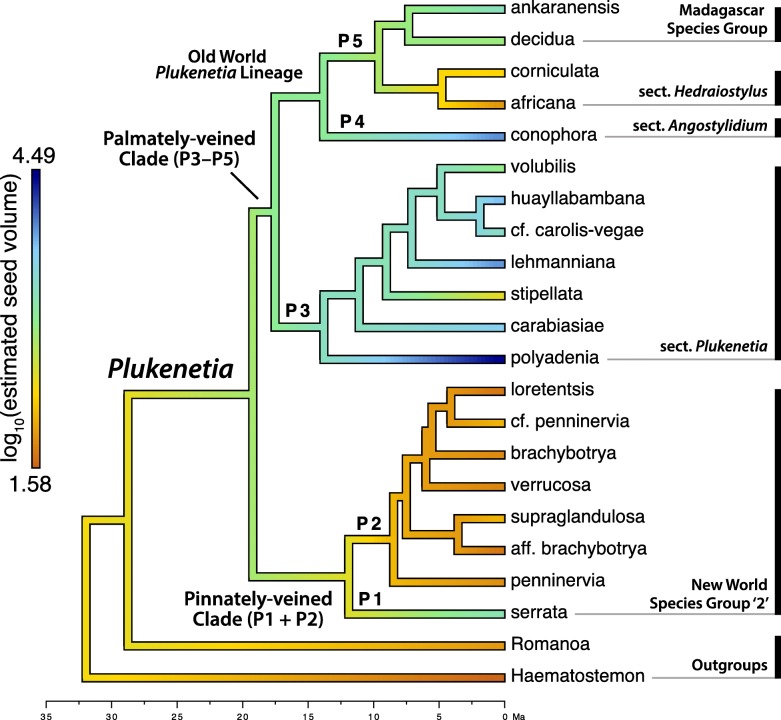
Ancestral state estimation of log_10_(estimated seed size volume) on the modified *Plukenetia* BEAST chronogram using the ContMap function of phytools

**Fig. 5:**
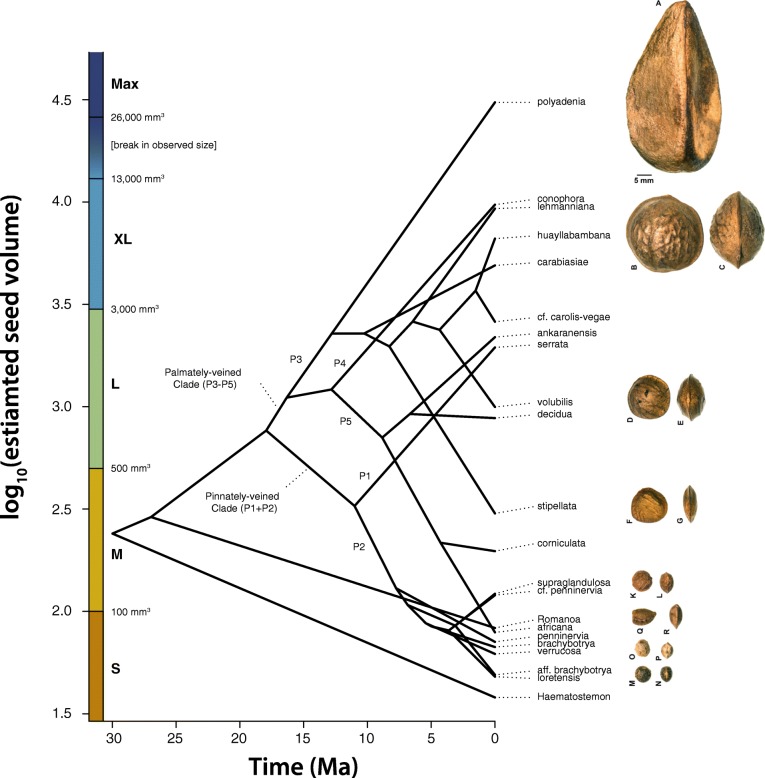
Traitgram of log_10_(estimated seed volume) mapped on the modified *Plukenetia* BEAST chronogram using the phenogram function of phytools. The colored scale on the left depicts seed size categories based on clustering analysis. On the right, representative seeds are shown in face and side view (see Fig. 1 for accession information)

Maximum likelihood continuous-character ancestral state estimation (Fig. 4) revealed the ancestor of Plukenetiinae most likely had M seeds, with S seeds becoming established in *Haematostemon* and *Romanoa*. The ancestor of *Plukenetia* likely had L seeds, followed by a transition to M seeds in the ancestor of the pinnately-veined clade (P1 + P2) while retaining L seeds in the ancestor of the palmately-veined clade (P3–P5). Within the pinnately-veined clade (P1 + P2), L seeds became fixed in *P. serrata* (P1) and S seeds in the ancestor of the remaining species of NWSG2 (P2). Section *Plukenetia* (P3) is characterized by four independent increases (XL: *P. carabiasiae*, *P. huayllabambana*, *P. lehmanniana*; Max: *P. polyadenia*) and one decrease (M: *P. stipellata*) from L seeded ancestors. The ancestor of the Old World *Plukenetia* lineage (P4 + P5) most likely had L seeds, with an inferred increase to XL in *P. conophora* (P4) and successive reductions to M and S–M seeds in *P. corniculata* and *P. africana*, respectively (P5). Traitgram analysis recovered similar results as ML ancestral state estimation and shows repeated divergences to larger and smaller seeds through time (Fig. 5).

### Correlates with seed size evolution

Phylogenetic regression under the threshold model indicate that seed size had moderate to strong positive relationships with plant size (*r* = 0.72), fruit type/dispersal mechanism (*r* = 0.66), and seedling ecology (*r* = 0.57), a weak negative association with fire tolerance (*r* = − 0.36), and negligible association with biome type (*r* = − 0.08) (Table 3). Plots of the posterior distribution for each coefficient (*r*) are available in Additional file 4: Figure S8.

**Table 3:** Results of phylogenetic regression analysis using the threshold model

Model	Coefficient (*r*) mean	Coefficient (*r*) 95% HPD
Seed size ~ Plant size	0.72	0.39 to 0.95
Seed size ~ Fruit type/dispersal mechanism	0.66	0.24 to 0.95
Seed size ~ Seedling ecology	0.57	0.09 to 0.96
Seed size ~ Biome	−0.08	−0.65 to 0.51
Seed size ~ Fire tolerance	−0.36	−0.87 to 0.31

## Discussion

### Low-copy nuclear gene identification

Our approach for identifying low-copy nuclear genes was effective but only moderately successful for our purposes. We recovered six promising low-copy genes, but the limited number of genomes/transcriptomes made it difficult to assess for lineage-specific paralogy until after sequencing. PCR amplification was challenging for low-copy nuclear genes compared to multiple-copy plastid and nuclear ribosomal DNA markers, particularly for low-yield DNA extractions common in tropical herbarium specimens, which was the vast majority of our sampling (81/86 accessions). Future applications of a top-down bioinformatics approach to marker development like the Lite Blue Devil pipeline should consider the potential limitations of DNA quality and if there are sufficient genome/transcriptome resources.

Our successful candidate was *KEA1*, a potassium (K+) efflux antiporter gene with putative function in maintaining K+ homeostasis and lowering osmotic potential [97]. It belongs to the cation/proton antiporter 2 (CPA2) supergene family, which includes the diverse cation-H+ exchanger (CHX) gene family [98]. The KEA gene family is highly conserved across green plants and is divided into three subtypes each comprising a variable number of genes [99]. Although unverified, *KEA1* is likely the only gene in the KEA-Ia subtype for *Ricinus communis, Plukenetia*, and by extension a majority of subfamily Acalyphoideae. At the species level, *KEA1* exons are highly conserved, therefore we designed primers for two introns that had higher nucleotide variation.

Outside of our genome/transcriptome data-mining pathway, we included the single-copy nuclear gene *TEB* for phylogenetic analysis. *TEB* encodes for the helicase and polymerase-containing protein TEBICHI, which is a putative plant homolog of mammalian DNA polymerase θ and has broad function in plant DNA repair and cell differentiation [100]. Little is known about the diversity and function of this gene in plants outside of *Arabidopsis*. Here we designed primers for a large and moderately variable exon, which is located outside of the functional helicase and polymerase domains of the gene.

### Phylogenetic relationships and systematic implications

Our results agree with the only other study examining the phylogenetic relationships of *Plukenetia* [6] but provide a more detailed and strongly supported hypothesis for species relationships in the genus. Here, *Plukenetia* is resolved into two main clades, the pinnately-veined clade (P1 + P2) comprising NWSG2, and the palmately-veined clade (P3–P5) containing sect. *Plukenetia* (P3) + the Old World *Plukenetia* lineage (P4 + P5).

The pinnately- (P1 + P2) and palmately-veined (P3–P5) clades overlap in androecium and gynoecium characters, but are differentiated by pollen tectum and leaf morphology. The pinnately-veined clade (P1 + P2) is equivalent to NWSG2, which was defined by Gillespie [1] to include species with “entirely connate styles, all or mostly sessile anthers, pollen with reticulate tectum, small dry capsules (except *P. serrata*), and elliptic pinnately-veined leaves (except *P. verrucosa*)”. Of those characters, pollen with reticulate tectum and elliptic pinnately-veined leaves are diagnostic features. The palmately-veined clade (P3–P5) contains the remaining four sections/species groups, sect. *Plukenetia*, sect. *Angostylidium*, sect. *Hedraiostylus*, and the Madagascan species group, of which the latter three compose the Old World lineage. Although species of the palmately-veined clade are morphologically diverse, especially in floral variation of the Old World lineage [2], they are united by foveolate pollen tectum and cordiform, ovate, or broadly elliptic leaves with palmate or triplinerved venation [1, 2]. Sect. *Plukenetia* has larger pollen grains (50.5–56 × 56.5–64.5 μm) than the Old World lineage (30.5–39 × 36.5–45.5 μm), although similar pollen size variation is found in the pinnately-veined clade [101].

Within the pinnately-veined clade (P1 + P2) we recovered novel support that *Plukenetia serrata* (P1) is sister to the remaining species of NWSG2 (P2). Cardinal-McTeague and Gillespie [6], using ITS and *psbA-trnH* with insertion/deletion gap-coded data, recovered subclades P1 and P2 in a functional polytomy with the palmately-veined clade (P3–P5), causing doubt over the monophyly of NWSG2. This study strongly supports the monophyly of a pinnately-veined clade (P1 + P2) and validates Gillespie’s [1] original hypothesis that reticulate pollen exine and pinnate leaf venation are synapomorphies for the lineage. Gillespie [1] also hypothesized that *P. verrucosa* was sister to the remainder of NWSG2 since it has plesiomorphic triplinerved leaves. However, here we recover *P. verrucosa* nested inside the pinnately-veined clade suggesting that its leaf morphology is a reversal to a more palmate-like condition.

Within the palmately-veined clade (P3–P5), we provide new evidence for a strongly-supported, monophyletic Old World lineage and backbone topology, where sect. *Angostylidium* (P4) is sister to sect. *Hedraiostylus* + the Madagascar species group (P5). In Cardinal-McTeague and Gillespie [6], the monophyly of the Old World lineage was not well-supported (PP = 0.87), which presented the possibility that sects. *Plukenetia* (P3) and *Angostylidium* (P4) could form a clade based on their similar leaf, floral, and fruit morphology [2]. Alternatively, our results suggest that the shared morphology of sects. *Plukenetia* (P3) and *Angostylidium* (P4) is plesiomorphic for the palmately-veined clade (P3–P5) and that the morphology of sect. *Hedraiostylus* and the Madagascar species (P5) is derived.

### Putative hybridization events in *Plukenetia*

An unexpected result of our study was evidence for two putative hybridization events between closely related species of *Plukenetia*. The first is a proposed ancient or recent hybridization event that resulted in the introgression of a *P. volubilis* plastid genome into both accessions of *P. huayllabambana* (*Téllez et al. 4*, *Quipuscoa 381*). *Plukenetia huayllabambana* is part of a high elevation species complex related to *P. volubilis*, which also comprises *P. carolis-vegae* and *P.* cf. *carolis-vegae*. *Plukenetia huayllabambana* and *P. carolis-vegae* were recently described from the Amazonas region of northern Peru and are noted for the economic significance of their cultivated oil-rich XL seeds [74, 75]. In contrast, *Plukenetia* cf. *carolis-vegae* appears to comprise non-cultivated populations with L seeds that are widespread in the Cusco, Junín, and Pasco regions of central and southern Peru. Members of the high elevation species complex share similar morphology but their distinguishing characters are breaking down as more collections become available (Table 4). Our data suggests that *P. huayllabambana* is a hybrid between *P.* cf. *carolis-vegae* and *P. volubilis* on the basis of plastome introgression (Additional file 4: Figure S3) and intermediate staminate floral morphology (Table 4). A paratype of *P. huayllabambana* (*Téllez et al. 4*, sampled here) possesses the introgressed plastome, which implies that the similar looking holotype was also based on hybrid material. Moving forward, we need to examine more material of the high elevation species complex from northern Peru, clarify if *P. carolis-vegae* is a naturally occurring or (semi-)domesticated species, and determine if it is distinct from the central/southern population of *P.* cf. *carolis-vegae*.

**Table 4:** Diagnostic characters of the high elevation species complex (*Plukenetia carolis-vegae*, *P.* cf. *carolis-vegae*, *P. huayllabambana*) and *P. volubilis*. Most characters were updated from species descriptions; † = unverified and potentially inaccurate

Character	*P. carolis-vegae* Bussmann, Paniagua & C.Téllez	*P.* cf. *carolis-vegae*	*P. huayllabambana* Bussmann, C.Téllez & A.Glenn [putative cf. *carolis-vegae* × *volubilis*]	*P. volubilis* L.
Sampled in phylogeny	No	Yes	Yes	Yes
Geographic range in Peru	Amazonas	Cusco, Junin, Pasco	Amazonas, Cajamarca	Amazonas, Cusco, Junin, Loreto, Madre de Dios, Pasco, San Martin
Elevation	1850 m	1450–2250 m	1200–2150 m	130–900(− 1800) m
Leaf stipels	1–2 thick stipels	1–2 thick stipels	1–2 thick stipels	1 knob (thick stipel)
Staminate sepals	4 (rarely 5) †	5 (sometimes 4 + 1 very thin, rarely 4)	4	4
Staminate nectaries	Absent †	Large irregularly shaped segments intermixed between stamens	Large irregularly shaped segments intermixed between stamens	Absent
Filament length	0.5–1 mm †	1–1.5 (2) mm	0.1–0.5 (1) mm	~ 0.5 mm
Style length	4–7 mm †	5–12 mm	6–7.5 mm	(9–)15–35 mm
Seed size measurements (length x width x thickness)	27 × 25 × 20 mm	19–20 × 17–18.6 × 13–15.8 mm	25–28 × 22–27 × 17–20 mm	13–22 × 11.8–18 × 5–9 mm
Seed size category	XL	L	XL	(M) L

The second event is less clear but we infer there may have been hybridization and/or incomplete lineage sorting within subclade P2 (Additional file 4: Figure S3). There are multiple weakly supported incongruences between the plastid and nuclear relationships of subclade P2, as well as a strongly supported incongruence in the placement of accession *Solomon 7972*. This specimen is morphologically attributed to *Plukenetia loretensis* (WCM, LJG, personal observations) but does not resolve with that species in either plastid or nuclear analyses. *Solomon 7972* is strongly supported as belonging to the *P. brachybotrya* clade in nDNA analyses, and is unresolved but strongly-supported outside of the *P. brachybotrya* clade by cpDNA. Chromosome number is variable within individuals of *P. volubilis* [102], and high and variable ploidy levels are suspected in other Plukenetieae genera (*Dalechampia* and *Tragia*) [103, 104]. Together this suggests that hybridization and possible allopolyploidization could be additional contributing factors to the incongruent relationships and evolutionary forces within *Plukenetia*.

### Neotropical biogeography and the origin of *Plukenetia*

The biogeographical history presented here is the first detailed analysis for *Plukenetia* and Plukenetiinae. Using secondary node calibrations based on subfamily age estimates [30], we find that Plukenetiinae and its genera diverged in the Oligocene (33.9 to 23.0 Mya) during the transitional period between the warm humid Eocene and the cooler drier Miocene [105]. By the Oligocene, the continents had already diverged and were well-separated by oceans and seas [106], precluding a Gondwanan vicariance explanation for the pantropical distribution of *Plukenetia*. BioGeoBEARS analyses indicate the ancestor of Plukenetiinae occupied a broad distribution comprising the Amazon and Atlantic Forest regions (Fig. 3) at a time when they likely formed a large continuous forest in the process of being divided by the open vegetation diagonal (now composed of the Chaco, Cerrado, and Caatinga biomes [32]). The open vegetation diagonal is an important feature of South American biogeography that acts as a barrier for moist forest plants and animals that cannot adapt and enter the drier biome conditions. The precise timing of the open vegetation diagonal’s formation is ambiguous but divergence dates between Amazon and Atlantic forest clades of suboscine birds [107], shield frogs [108], and spectacled lizards [109] suggest the barrier was present by the Oligocene and Early Miocene. Yet, some plant lineages did not diversify in these open vegetation biomes until the Late Miocene and Pliocene, as is suggested by a Late Miocene radiation of orchids in the Campos Rupestres (rocky savannas) [110] and a Pliocene radiation of several fire-adapted genera (including *Mimosa*) in the Cerrado [111]. Our data agree with an Oligocene origin for the open vegetation diagonal barrier (Fig. 3), which suggests there was a putative transitional stage between when the open vegetation diagonal barrier formed (Oligocene to Middle Miocene) and when modern Chaco, Cerrado, and Caatinga communities developed (Late Miocene to Pliocene).

*Plukenetia* and its sister genus, *Romanoa*, are suggested to have diverged in the Atlantic Forest, presumably after it was isolated from the Amazon (Fig. 3). *Romanoa* is estimated to have remained in the Atlantic Forest, while the common ancestor of *Plukenetia* either remained in the Atlantic Forest or migrated into the Amazon. Either scenario would invoke two dispersals or migrations between the Amazon and Atlantic Forests, which suggests that there were periodic connections across the open vegetation biogeographical barrier that allowed for biotic exchange. The marine ingression by the Paranaense Sea into northern Argentina, Paraguay, and southern Brazil [112] may have facilitated a forest connection along its coast during the Middle and Late Miocene (originally proposed by Costa [113]). The timing of Paranaense Sea coincides with the ancestor of subclade P2 migrating from the Atlantic Forest into the Amazon (Fig. 3). Older migrations during the Oligocene and Early Miocene do not have geological explanations and must invoke short distance dispersals or migrations across a putative mosaic of wet forest fragments. Similar explanations exist for Pliocene and Pleistocene crossings [107, 113]. Further examination of plant groups that exhibit an Amazon-Atlantic Forest disjunction [114] could shed additional light onto the formation of the open vegetation diagonal and the timing of past biotic exchanges. We could also investigate when neighbouring wet forest plants shifted into the seasonally dry biome of the open vegetation diagonal and converge on a general pattern of its history and formation.

Following entry into the Amazon, a common biogeographical pattern of New World *Plukenetia* lineages was their repeated dispersal across the Andes mountain barrier and beyond into Central America and Mexico. The first transition occurred in subclade P3 (after the divergence of *P. polyadenia*) during the Middle Miocene (Fig. 3). At this time the Isthmus of Panama was not fully formed [115], implying a short-distance dispersal between South and Central America. The second occurred in subclade P2 in the ancestor of *P. penninervia* during the Late Miocene. These patterns agree with previous indications that many plant lineages dispersed between Central and South America prior to the formation of the Isthmus of Panama [116–118].

A dispersal event across the Andes and back into the Amazon is revealed in the ancestor of *Plukenetia* cf. *carolis-vegae* and *P. volubilis* during the Pliocene (Fig. 3). The timing of re-entry into the Amazon coincides with the highest uplift of the Andes and a period of rapid plant diversification along elevational gradients [115]. *Plukenetia* did not rapidly diversify in response to the uplift of the Andes, but adaptation to either low-to-medium (100–800 m; *P. volubilis*) or high (1500–2500 m; *P.* cf. *carolis-vegae*) elevation environments likely contributed to the speciation of those taxa. A second dispersal across the Andes, from the Amazon into northwestern South America, is inferred in *P.* cf. *penninervia* during the Pliocene (Fig. 3). Together, our data supports increasing evidence that tropical plants dispersed across the Andes mountains after achieving their maximum elevation in the Pliocene [31].

### Trans-oceanic dispersals explain the pantropical distribution of *Plukenetia*

The pantropical distribution of *Plukenetia* is best explained by multiple trans-oceanic dispersal events throughout the Miocene and Pliocene (Fig. 3). The ancestor of the Old World lineage likely underwent a trans-Atlantic Ocean crossing from the Amazon to tropical Africa during the Early Miocene. Current hypotheses suggest recent South American and African disjunctions are the result long-distance dispersal by trade winds or tangled plant mats crossing the Atlantic Ocean on equatorial currents [119]. *Plukenetia* most likely dispersed by water along the north equatorial countercurrent, and joins the growing list of taxa that dispersed in the less-frequently inferred direction from South America to Africa during the Miocene, e.g., *Pitcairnia* (Bromeliaceae) [120, 121], Melastomeae (Melastomataceae) [85, 122, 123], *Vanilla* (Orchidaceae) [87], *Maschalocephalus* (Rapateaceae) [120], and *Erismadelphus* (Vosychiaceae) [85, 124].

We infer two further dispersal events from Africa into Madagascar and Southeast Asia within *Plukenetia* subclade P5 (Fig. 3). The Madagascar species group diverged in the Late Miocene and likely experienced a short-distance dispersal event across the Mozambique channel from Africa, at a time when once widespread mainland rainforests were transitioning to woodlands and savanna [125]. Increasing molecular evidence suggests plant diversity in Madagascar has been influenced by multiple arrivals from Africa during and since the Miocene, e.g., *Uvaria* (Annonaceae) [126], *Boscia*, *Cadaba*, *Thilachium* (Capparaceae) [86], Cyatheaceae [127], Hibisceae (Malvaceae) [128], Rubiaceae [129], and *Rinorea* (Violaceae) [130].

The ancestor of *Plukenetia corniculata* most likely underwent a trans-Indian Ocean long-distance dispersal from Africa into Southeast Asia in the Pliocene (Fig. 3). Africa-to-Asia long-distance dispersals are still poorly understood but are indicated for several taxa starting from the Oligocene, including *Begonia* (Begoniaceae) [131], *Exacum* (Gentianaceae) [132], *Osbeckia* (Melastomataceae) [133], *Eurycoma* (Simaroubaceae) [134], and *Cissus* (Vitaceae) [135]. The Pliocene divergence of *P. corniculata* post-dates migration or step-wise dispersal via the Indian subcontinent or Eocene boreotropical forest, which emphasizes the probable role of the Indian Ocean equatorial countercurrent in transporting tangled plant mats from Africa to Southeast Asia [126, 136].

### Seed size classification and the origin of large seeds

Clustering analysis of seed dimension data provided a more objective and finely parsed seed size classification for *Plukenetia* (Table 2). The prior subjective “small versus large” informal groupings have been replaced with five discrete categories that additional seeds can be measured and compared against. The former “small” category is divided into S and M seeds, while the “large” category now comprises L, XL, and Max seeds.

Our new seed size classification allows us to present a more nuanced interpretation of seed evolution in *Plukenetia* (Figs. 4 and 5). We found pronounced seed size differences in both the pinnately-veined (P1 + P2) and palmately-veined (P3–P5) clades, with more variation in the latter. Our results suggest that seed size evolution can be dynamic and responsive to ecological selection, although phylogenetically conserved in some cases (e.g., pinnately-veined subclade P2). The rest of tribe Plukenetieae is fairly uniform in having S to M seeds (data not shown), suggesting usually strict genetic controls of seed size have been relaxed in *Plukenetia*.

The ancestor of *Plukenetia* is estimated as having L seeds, suggesting there was a single origin in the genus. Under this scenario, there was likely a reduction to M seeds in the ancestor of the pinnately-veined clade (P1 + P2) followed by continued size reduction in NWSG2 (P2) and a return to L seeds in *P. serrata* (P1) (Fig. 4). The molecular mechanisms controlling seed size are not known in *Plukenetia* but could be studied by comparing seed transcriptomes between divergent sister group pairings.

The ancestor of the palmately-veined clade (P3–P5) most likely had L seeds, which suggests that XL seeds evolved independently up to five times in sects. *Plukenetia* (P3) and *Angostylidium* (P4). Max seeds evolved once in the ancestor of *P. polyadenia*, the earliest diverging lineage of sect. *Plukenetia* (P3). *Plukenetia polyadenia* seeds are substantially larger than all other species (Fig. 1a) and are suggested to have evolved over a long period of time (since the Middle Miocene) in the Amazon. This species appears especially adapted for low light seedling establishment and uses considerable stored reserves to send out a long leafless leader (LJG, KJW, personal observations). The size, shape, and colour of *P. polyadenia* fruits and seeds are compatible with an extinct South American megafauna dispersal syndrome [137], although its widespread distribution contradicts expected range reduction following the loss of such dispersers.

### Multiple traits correlated with seed size evolution

Our study presents, to our knowledge, one of the first analyses of seed size variation across a small, densely-sampled phylogenetic lineage (~ 23 species). While trends in seed size evolution have been studied within species [13–15] and on a macroevolutionary scale [8–11], evolutionary patterns among groups of species have not been well documented. Studies on entire clades allow us to understand the context in which seed size variation develops, as well as directs future comparative research on seed ecology and genetics.

We found that seed size in *Plukenetia* is associated with a combination of traits (plant size, fruit type/dispersal mechanism, seedling ecology), which suggests that the evolution of substantial seed size variation relied on multiple selective pressures rather than a single driving force. By comparison, a study of *Hakea* (Proteaceae), a diverse group of 150 species of small trees and shrubs that largely occur in fire-prone ecosystems in southwestern Australia, found that seed size was correlated with a different set of traits (fecundity/seed production and postfire regeneration) [17]. As more clade-based studies emerge we will be able to identify common trends in seed size evolution and how they relate or differ across growth forms and habitats.

### Seed size trends in *Plukenetia*

Within *Plukenetia* we observe a strong association between smaller seeds in herbaceous vines and slender lianas up to the largest seeds in thick stemmed canopy lianas (Table 5). Our results are consistent with broader trends in seed plants in which plant size and seed mass are strongly correlated, more so than temperature, forest cover, and dispersal mechanism [138]. One explanation is that larger plants require more time to reach reproductive maturity, which could drive selection for larger seeds with increased survival rates to reproductive age [139]. However, *Plukenetia* species start reproducing within one or two years regardless of plant size, suggesting that other ecological factors are driving seed and plant size. Furthermore, while smaller plants may produce more seeds early on, larger plants tend to occupy more canopy space and live longer, resulting in largely equivocal total lifetime seed production [140]. Comparative analysis of plant size, seed mass, lifespan, and total seed production among *Plukenetia* species with different seed sizes could shed more light on the association between seed and plant size.

**Table 5:** Traits associated with seed size for species of *Plukenetia* and outgroup genera (arranged by size). DDF = dry deciduous forest; ERF = evergreen rain forest; SMF = seasonal moist forest

Species/ species group	Sub-clade	Biome	Habitat	Growth form	Stem size	Fruit type	Seed size category	Seedling ecology
*P. polyadenia*	P3	ERF	Lowland forest; riparian forest	Canopy liana	Thick	Fleshy	Max	Shade
*P. conophora*	P4	ERF, SMF	Lowland forest	Canopy liana	Thick	Fleshy	XL	Shade
*P. lehmanniana*	P3	ERF, SMF	Lowland to montane forest edges, light gaps	Slender to robust liana	Thick	Fleshy	XL	Light gap
*P. huayllabambana*	P3	ERF, SMF	Montane forest edges, ravines, light gaps	Slender to robust liana	Thick	Dry	XL	Light gap
*P. carabiasiae*	P3	ERF	Montane forest	Canopy liana	Thick	Dry	XL	Shade
*P.* cf*. carolis-vegae*	P3	ERF, SMF	Montane forest edges, ravines, light gaps	Slender to robust liana	Thick	Dry	XL	Light gap
*P. serrata*	P1	SMF	Pre-montane to montane forest edges	Slender to robust liana	Thick	Fleshy	L	Light gap
Madagascan species	P5	DDF	Forest on limestone or sand; spiny forest scrub	Robust liana	Thick	Dry	L	Light gap
*P. volubilis*	P3	ERF, SMF, (semi-humid savanna)	Lowland to pre-montane forest edges, ravines, light gaps (seasonally flooded savanna woodland)	Vine to slender liana	Thin	Dry	(M) L	Light gap
*P. stipellata*	P3	ERF, SMF	Lowland forest edges, light gaps	Vine to slender liana	Thin	Dry	M	Light gap
*P. corniculata*	P5	ERF, SMF	Lowland forest edges, light gaps	Vine to slender liana	Thin	Dry	M	Light gap
*P. supraglandulosa*	P2	SMF	Lowland to submontane forest edges, light gaps	Vine to slender liana	Thin	Dry	M	Light gap
*P.* cf. *penninervia*	P2	EMF	Lowland pluvial forest edges, light gaps	Vine to slender liana	Thin	Dry	M	Light gap
*P. africana*	P5	Semi-arid savanna	Savanna woodland on sandy soil	Vine with thick rootstock	Thin	Dry	S–M	Light gap
NWSG2 pro parte (excluding *P.* cf. *penninervia*, *P. serrata*, *P. supraglandulosa*)	P2	ERF, SMF, (DDF)	Lowland to pre-montane (montane) forest edges, rocky outcrops, light gaps; white sand forest	Vine to slender liana	Thin	Dry	S	Light gap
*Romanoa*	n/a	SMF	Semi-deciduous lowland forest, rocky outcrops, light gaps; stony soil caatinga	Vine to slender liana	Thin	Dry	S	Light gap
*Haematostemon*	n/a	ERF	Lowland forest; riparian forest	Small tree/ shrub	Thin	Dry	S	Light gap

Most *Plukenetia* species inhabit evergreen and seasonally moist forests, where seed size was likely driven by competing selection among plant size, dispersal mechanism, and seedling ecology. Although moist forest species of *Plukenetia* are widespread, they do not form a dominant component of forest vegetation or fit into early successional communities. Rather, they form a small but common element of primary and secondary forest edges and light gaps associated with treefall disturbances and natural light breaks from rivers and rocky outcrops (Table 5). Moist forest *Plukenetia* seeds have a high probability of being dispersed into shaded areas so there should be a trade-off between producing many smaller seeds that have a higher probability of being dispersed into favourable light gaps, and fewer larger seeds that may not be dispersed far but produce more resilient seedlings that can tolerate or avoid low light conditions [141]. The largest *Plukenetia* seeds are associated with canopy liana species (i.e., *P. conophora*, *P. polyadenia*), which have the added challenge of fueling seedling growth upwards to reach as much light as possible in the understory. It seems likely that movement into different niche spaces (i.e., light gap versus canopy) is the driving force of seed size extremes in *Plukenetia*, and that variation therein is a result of a balance between those competing selective pressures.

Biome shifts were not correlated with seed size changes, except in the transition to semi-arid savannas in *Plukenetia africana*. In this case, increasing aridification, forest fragmentation, seasonality, and fire regimes [125] are thought to have selected for smaller plants with perennial woody rootstocks and short-lived seasonal stems [2]. This smaller seasonal growth form is better adapted to survive prolonged dry seasons and facilitates resprouting after fires [142]. We recovered a weak negative association between fire tolerance and seed size, but with a sample size of one it is difficult to draw a strong conclusion. Furthermore, while there is typically a positive association between increased seed size and embryo survival in fire-prone systems [23, 143, 144], seed size can be constrained by conflicting selective pressure from seed predation [13, 143] or by a close association with plant size as is suggested by our data. We note that traits other than seed size can compensate for survival in fire-prone systems, such as seed pubescence, shape, and pericarp thickness [145], although these do not appear of relevance in *P. africana*.

Dispersal syndromes in *Plukenetia* are not yet well-documented but can be predicted based on fruit colour, dehiscence, and seed size and content. Species with S and M seeds usually have dry brown capsules that explosively dehisce and release their seeds to the ground below within a few meters of the parent. This fruit type is typical for Euphorbiaceae in general and is likely plesiomorphic in the genus. Assuming that all *Plukenetia* species have seeds with high fatty acid content, we hypothesize that S and M seeds would be secondarily dispersed by scatter hoarding rodents that preferentially search for valuable high energy seeds [26, 27]. Scatter hoarding is a reliable short distance dispersal mechanism in neotropical forests [25], which could favour smaller seeds since larger seeds are more likely to be preyed upon [13]. *Plukenetia* species with XL and Max seeds often have green or brown, thinly fleshed, indehiscent berries. These large, high energy fruits could be part of the diverse diet of larger mammals such as primates [28, 29]. If dispersed by large mammals, we suspect that a majority of XL and Max seeds would be heavily preyed upon and inefficiently dispersed, although occasionally they could be transported further distances than possible by scatter hoarding rodents. Some L and XL seeds are intermediate and have semi-indehiscent, somewhat fleshy capsules that dry slowly and eventually dehisce to disperse their seeds. In this case, they are possibly dispersed by a combination of scatter hoarding and larger mammal syndromes.

## Conclusions

Here, we identified two novel low-copy nuclear genes for phylogenetic analysis (*KEA1* and *TEB*), resolved the backbone and a majority of the species relationships in *Plukenetia*, and produced a robust chronogram for time-dependent evolutionary analysis of the genus. We found support for the monophyly of two major clades, the pinnately-veined clade (P1 + P2) composed of NWSG2, and the palmately-veined clade (P3–P5) composed of sects. *Plukenetia* (P3), *Angostylidium* (P4), and *Hedraiostylus* + the Madagascar species group (P5). Molecular dating and biogeographical analyses suggest that *Plukenetia* originated in either the Amazon or Atlantic Forest of Brazil during the Oligocene. The early biogeographical history of *Plukenetia* is equivocal between these two areas, but suggests a general trend of migration across the open vegetation diagonal during the Oligocene and Early to Mid Miocene. Ancestors in the Amazon underwent at least two independent dispersals into Central America and Mexico prior to the formation of the Isthmus of Panama in the Mid and Late Miocene. The ancestor of *P. volubilis* and *P.* cf. *carolis-vegae* was the only lineage to return to the Amazon from Central and NW South America, by an inferred dispersal over the Andes during the Pliocene. The pantropical distribution of *Plukenetia* is best explained by trans-oceanic long-distance dispersals, first to Africa in the Early Miocene and then independently to Madagascar and Southeast Asia, during the Late Miocene and Pliocene. We estimate that there was a single origin of L seeds in the ancestor of *Plukenetia*. Within *Plukenetia*, seed size evolution is dynamic and correlated with plant size, fruit type (including inferred dispersal mechanism), and seedling ecology. Biome shifts were not associated with seed size, however, the transition to a seasonal, fire-regimented savanna recovered a weak association with seed size reduction. Quantitative seed ecology studies are needed to elaborate on the trends we identified in *Plukenetia*, and would serve as groundbreaking clade-based investigations into the drivers of seed size variation.

## Additional files

Additional file 1 **Table S1.** Accession table for vouchers used in phylogenetic analyses. **Table S2.** Accession table for genomes/transcriptomes used in data-mining analyses. **Table S3.** List of molecular markers, primer sequence, and amplification/sequencing protocols. **Table S4.** Biogeographical distribution matrix. **Table S5.** Manual dispersal matrix between biogeographical areas. **Table S6.** Trait matrix used in phylogenetic regression analysis with seed size. **Table S7.** The best evolutionary model for seed size based on ΔAIC and Akaike weights (*w*_*i*_). (PDF 257 kb)

Additional file 2 Python script for Lite Blue Devil v0.3. (PY 12 kb)

Additional file 3 Setting file for Lite Blue Devil v0.3. (TXT 835 bytes)

Additional file 4 **Figure S1.** Primer map for new (*KEA1* introns 11 and 17, *TEB* exon 17) and redesigned (ETS, *matK*) molecular markers. **Figure S2.** Shortest maximum likelihood tree for incongruence analysis of individual markers: (a) ETS, (b) ITS, (c) *KEA1* intron 11, (d) *KEA1* intron 17, (e) *TEB* exon 17, (f) *matK*, and (g) *ndhF*. Bootstrap percentages based on 500 replicates. Well-supported branches (> 85% maximum likelihood bootstrap percentage; MLBP) are in bold. **Figure S3.** Bayesian maximum clade credibility tree based on (a) plastid DNA (cpDNA) two marker, 74 accession dataset, and (b) nuclear DNA (nDNA) five marker, 86 accession dataset, for *Plukenetia* and Plukenetiinae outgroups. Maximum parsimony bootstrap percentage (MPBP) and Bayesian posterior probability (PP) support values > 50% are indicated on each branch. Branches in bold indicate strong support (≥ 85 MPBP and ≥ 0.95 PP). Grey boxes highlight strongly supported topological incongruences. **Figure S4.** BEAST chronogram of *Plukenetia* and Plukenetiinae outgroups inferred from the combined seven marker (cpDNA and nDNA), 83 accession dataset and two normal-distribution priors (indicated in red) based on previous subfamily Acalyphoideae estimates using three fossil calibrations. Numbers at each node indicate mean age estimates, and blue bars the 95% highest posterior density confidence interval. **Figure S5.** BioGeoBEARS ancestral range estimation probabilities for each corner and node under (a) DEC + J and (b) DEC. **Figure S6.** UPGMA clustering analysis of log_10_ transformed seed dimensions (length, width, thickness) for 190 accessions of *Plukenetia*. **Figure S7.** Principal components analysis of log_10_ transformed seed dimensions (length, width, thickness) for 190 accessions of *Plukenetia*. **Figure S8.** Posterior distributions of the correlation coefficients (*r*) between the liabilities of seed size and (a) plant size, (b) fruit type, (c) seedling ecology, (d) fire tolerance, and (e) biome type, under the threshold model. (PDF 2520 kb)

Additional file 5 Seed size measurements for *Plukenetia* and Plukenetiinae outgroups. (CSV 15 kb)

## Abbreviations

+J: Founder-event parameter
AddMRCA: Add most recent common ancestor
AIC: Akaike information criterion; ARE: ancestral range estimation
CAY: Herbier de Guyane
CHX: Cation-H+ exchanger
CPA2: Cation/proton K+ antiporter 2
cpDNA: Plastid DNA
DDF: Dry deciduous forest
DEC: Dispersal-extinction-cladogenesis
ERF: Evergreen rain forest
ESS: Effective sample size
ETS: Nuclear ribosomal DNA external transcribed spacer region
Exo: Exonuclease I
exp.: Exponential
HPD: Highest posterior density
ITS: Nuclear ribosomal DNA internal transcribed spacer region
*KEA1*: Potassium (K+) efflux antiporter nuclear gene
L: Large
lnL: Log likelihood
M: Medium
*matK*: Maturase K plastid gene
Max: Maximum
MCMC: Markov chain Monte Carlo
ML: Maximum likelihood
MLBP: Maximum likelihood bootstrap percentage
MO: Missouri Botanical Garden Herbarium
MPBP: Maximum parsimony bootstrap percentage
Mya: Million years ago
*ndhF*: NADH dehydrogenase F plastid gene
nDNA: Nuclear DNA
NWSG2: New World species group “2”
ORF: Open reading frame
P1–P5: A subclade naming system for Plukenetia
PC1: First principal component
PCA: Principal components analysis
PERMANOVA: Permutated analysis of variance
PP: Posterior probability
PSRF: Potential scale reduction factor
rjhp: Reversible-jump hyperprior
rjMCMC: Reversible-jump Markov chain Monte Carlo
S: Small
SAP: Shrimp alkaline phosphatase
SD: Standard deviation
SMF: Seasonal moist forest
TBR: Tree-bisection-reconnection
*TEB*: Helicase and polymerase-containing protein TEBICHI nuclear gene
TMRCA: Time to most recent common ancestor
UCLN: Uncorrelated lognormal
UPGMA: Unweighted pair-group method using arithmetic averages
US: United States National Herbarium
*w*_*i*_: Akaike weights
XL: Extra-large
θ: Theta
