# Supplementary material for: Seed size evolution and biogeography of Plukenetia (Euphorbiaceae), a pantropical genus with traditionally cultivated oilseed species
Source: BMC Evol Biol. 2019 Jan 22;19:29. doi: 10.1186/s12862-018-1308-9 (PMC6341577; doi:10.1186/s12862-018-1308-9)
Supplement: Supplementary file 1 — Table S1. Accession table for vouchers used in phylogenetic analyses. Table S2. Accession table for genomes/transcriptomes used in data-mining analyses. Table S3. List of molecular markers, primer sequence, and amplification/sequencing protocols. Table S4. Biogeographical distribution matrix. Table S5. Manual dispersal matrix between biogeographical areas. Table S6. Trait matrix used in phylogenetic regression analysis with seed size. Table S7. The best evolutionary model for seed size based on ΔAIC and Akaike weights (wi). (PDF 257 kb) [file 12862_2018_1308_MOESM1_ESM.pdf]

Table S1. Accession table for vouchers used in phylogenetic analyses (bolded text indicates new GenBank accessions).

No.	Species	Voucher	Country	ITS	ETS	*KEA1* intron 11	*KEA1* intron 17	*TEB* exon 17	*matK*	*ndhF*
1	*Haematostemon guianensis* Sandwith	Wurdack 4350 (US) [a]	Guyana	KP794434	**MF502427**	—	—	**MF502846**	**MF502706**	**MF502776**
2	*Plukenetia africana* Sond.	Bartsch 1859 (US)	Namibia	**MF502513**	**MF502431**	**MF502579**	**MF502651**	**MF502848**	**MF502710**	**MF502780**
3	*P. africana*	Guy 2367 (MO)	Botswana	**MF502514**	—	—	—	—	—	**MF502781**
4	*P. africana*	Palomoti 1086 (MO)	Botswana	**MF502515**	**MF502432**	—	—	—	**MF502711**	**MF502782**
5	*P. africana*	Pope et al. 834 (MO)	Botswana	**MF502516**	**MF502433**	**MF502580**	—	—	**MF502712**	**MF502783**
6	*P. ankaranensis* L.J.Gillespie	Gillespie et al. 10697 (CAN) [a]	Madagascar	KP794438	**MF502434**	**MF502581**	**MF502652**	**MF502849**	**MF502713**	**MF502784**
7	*P. ankaranensis*	Lees s.n. (CAN)	Madagascar	KP794437	**MF502435**	—	**MF502653**	**MF502850**	**MF502714**	**MF502785**
8	*P. brachybotrya* Müll.Arg.	Acevedo-Rodriguez 14416 (NY)	Peru	**MF502517**	**MF502436**	**MF502582**	**MF502654**	**MF502851**	**MF502715**	**MF502786**
9	*P. brachybotrya*	Araujo-M. et al. 1722 (MO)	Bolivia	KP794452	**MF502437**	**MF502583**	**MF502655**	**MF502852**	**MF502716**	**MF502787**
10	*P. brachybotrya*	Fuentes & Torrico 5398 (MO)	Bolivia	**MF502518**	**MF502438**	**MF502584**	—	—	**MF502717**	**MF502788**
11	*P. brachybotrya*	Galiano et al. 6612 (MO)	Peru	**MF502519**	**MF502439**	—	—	—	**MF502718**	**MF502789**
12	*P. brachybotrya*	Seidel & Vaquiata 7733 (MO)	Bolivia	**MF502520**	**MF502440**	**MF502585**	**MF502656**	**MF502853**	**MF502719**	**MF502790**
13	*P. aff. brachybotrya*	Ledezma et al. 921 (CAN)	Bolivia	**MF502511**	**MF502428**	**MF502576**	**MF502649**	—	**MF502707**	**MF502777**
14	*P. aff. brachybotrya*	Sperling et al. 5873 (MO)	Brazil	**MF502512**	**MF502429**	**MF502577**	**MF502650**	**MF502847**	**MF502708**	**MF502778**
15	*P. aff. brachybotrya*	Sperling et al. 6161 (CAN)	Brazil	—	**MF502430**	**MF502578**	—	—	**MF502709**	**MF502779**
16	*P. carabiasiae* J.Jiménez Ram.	Meave et al. 1550 (MO)	Mexico	**MF502521**	**MF502441**	**MF502586**	—	—	**MF502720**	—
17	*P. cf. carolis-vegae*	Calatayud et al. 2643 (CAN)	Peru	**MF502527**	**MF502448**	**MF502592**	—	—	—	—
18	*P. cf. carolis-vegae*	Huamantupa et al. 6445 (CAN)	Peru	**MF502528**	**MF502449**	**MF502593**	**MF502660**	—	**MF502724**	—
19	*P. cf. carolis-vegae*	Monteagudo et al. 14125 (MO)	Peru	**MF502529**	**MF502450**	**MF502594**	**MF502661**	—	**MF502725**	**MF502793**
20	*P. cf. carolis-vegae*	Monteagudo et al. 15252 (MO)	Peru	**MF502530**	**MF502451**	—	—	—	**MF502726**	**MF502794**
21	*P. cf. carolis-vegae*	Rojas et al. 3863 (CAN)	Peru	**MF502532**	**MF502453**	**MF502595**	—	—	—	—

22	*P. cf. carolis-vegae*	Valenzuela et al. 5197 (CAN)	Peru	**MF502534**	**MF502455**	**MF502597**	**MF502663**	—	—	—
23	*P. cf. carolis-vegae*	van der Werff et al. 17532 (CAN)	Peru	**MF502535**	**MF502456**	**MF502598**	**MF502664**	**MF502856**	**MF502729**	**MF502797**
24	*P. cf. carolis-vegae*	Vasquez et al. 33145 (MO)	Peru	**MF502536**	**MF502457**	**MF502599**	**MF502665**	**MF502857**	**MF502730**	**MF502798**
25	*P. cf. carolis-vegae*	Woytkowski 6670 (MO)	Peru	**MF502537**	**MF502458**	**MF502600**	**MF502666**	**MF502858**	**MF502731**	**MF502799**
26	*P. conophora* Müll.Arg.	Hart 1621 (MO)	Democratic Republic of the Congo	**MF502523**	**MF502443**	**MF502588**	—	—	—	—
27	*P. conophora*	Nemba & Thomas 434 (MO)	Cameroon	KP794457	**MF502444**	**MF502589**	**MF502657**	—	**MF502722**	**MF502792**
28	*P. corniculata* Sm.	Huq & Haroon 10780 (GH)	Bangladesh	**MF502524**	**MF502445**	**MF502590**	**MF502658**	**MF502854**	—	—
29	*P. corniculata*	Huq & Haroon 10780 (MO)	Bangladesh	**MF502525**	**MF502446**	**MF502591**	**MF502659**	**MF502855**	**MF502723**	—
30	*P. decidua* L.J.Gillespie	Rakotomalaza 597 (CAN)	Madagascar	**MF502526**	**MF502447**	—	—	—	—	—
31	*P. huayllabambana* Bussmann, C.Tellez & A.Glenn	Quipuscoa 381 (MO)	Peru	**MF502531**	**MF502452**	—	—	—	**MF502727**	**MF502795**
32	*P. huayllabambana*	Tellez et al. 4 (MO)	Peru	**MF502533**	**MF502454**	**MF502596**	**MF502662**	—	**MF502728**	**MF502796**
33	*P. lehmanniana* (Pax & K.Hoffm.) Huft & L.J.Gillespie	Acevedo-Rodriguez & Daly 1658 (MO)	Ecuador	KP494443	**MF502459**	**MF502601**	**MF502667**	**MF502859**	**MF502732**	**MF502800**
34	*P. lehmanniana*	Clark 3953 (MO)	Ecuador	**MF502538**	**MF502460**	**MF502602**	**MF502668**	**MF502860**	**MF502733**	**MF502801**
35	*P. lehmanniana*	Silverstone-Sopkin & Giralda-Gesini 8019 (MO)	Colombia	**MF502539**	**MF502461**	**MF502603**	—	**MF502861**	**MF502734**	**MF502802**
36	*P. lehmanniana*	Zak & Jaramillo 3401 (MO)	Ecuador	**MF502540**	**MF502462**	**MF502604**	**MF502669**	**MF502862**	**MF502735**	**MF502803**
37	*P. loretensis* Ule	Grandez 19608 (AMAZ) [a]	Peru	KP794453	**MF502463**	**MF502605**	**MF502670**	**MF502863**	**MF502736**	**MF502804**
38	*P. loretensis*	McDaniel & Rimachi 22451 (MO)	Peru	**MF502541**	**MF502464**	**MF502606**	—	—	**MF502737**	**MF502805**
39	*P. loretensis*	Rimachi 5122 (MO)	Peru	**MF502542**	**MF502465**	**MF502607**	—	—	**MF502738**	**MF502806**
40	*P. loretensis*	Solomon 7972 (MO)	Bolivia	**MF502543**	**MF502466**	**MF502608**	—	**MF502864**	**MF502739**	**MF502807**
41	*P. loretensis*	Vásquez & Jaramillo 3283 (MO)	Peru	**MF502544**	**MF502467**	**MF502609**	—	—	—	—
42	*P. loretensis*	Vasquez et al. 38069 (MO)	Peru	**MF502545**	**MF502468**	**MF502610**	**MF502671**	**MF502865**	**MF502740**	**MF502808**
43	*P. madagascarensis* Leandri	Andrianjafy 1648 (CAN)	Madagascar	**MF502546**	**MF502469**	**MF502611**	**MF502672**	—	**MF502741**	—

44	*P. madagascarensis*	Gillespie 4175 (CAN)	Madagascar	**MF502547**	**MF502470**	**MF502612**	**MF502673**	**MF502866**	**MF502742**	**MF502809**
45	*P. madagascarensis*	Villiers et al. 4899 (MO)	Madagascar	**MF502548**	**MF502471**	**MF502613**	**MF502674**	**MF502867**	**MF502743**	**MF502810**
46	*P. penninervia* Müll.Arg.	Atha et al. 1001 (MO)	Belize	KP794455	**MF502472**	**MF502614**	**MF502675**	**MF502868**	**MF502744**	**MF502811**
47	*P. penninervia*	Carnevali & Duno 7594 (MO)	Mexico	**MF502549**	**MF502473**	**MF502615**	**MF502676**	**MF502869**	**MF502745**	**MF502812**
48	*P. penninervia*	Martínez 10527 (MO)	Mexico	KP794456	**MF502474**	**MF502616**	**MF502677**	**MF502870**	**MF502746**	**MF502813**
49	*P. penninervia*	Martinez 17705 (DAV)	Mexico	**MF502550**	**MF502475**	**MF502617**	**MF502678**	**MF502871**	**MF502747**	**MF502814**
50	*P. penninervia*	McPherson 8461 (MO)	Panama	KP794454	**MF502476**	**MF502618**	**MF502679**	**MF502872**	**MF502748**	**MF502815**
51	*P. penninervia*	Wallnöfer & Frisch 5996 (MO)	Guatemala	**MF502551**	**MF502477**	**MF502619**	—	**MF502873**	**MF502749**	**MF502816**
52	*P. cf. penninervia*	Gentry 47799 (MO)	Colombia	**MF502522**	**MF502442**	**MF502587**	—	—	**MF502721**	**MF502791**
53	*P. polyadenia* Müll.Arg.	Croat 20285 (MO)	Peru	**MF502552**	—	—	—	—	**MF502750**	**MF502817**
54	*P. polyadenia*	Gillespie 4314 (CAN)	Smithsonian Greenhouse ex French Guiana	**MF502553**	**MF502478**	**MF502620**	**MF502680**	**MF502874**	**MF502751**	**MF502818**
55	*P. polyadenia*	Wurdack 5288 (US)	Guyana	**MF502554**	**MF502479**	**MF502621**	**MF502681**	**MF502875**	**MF502752**	**MF502819**
56	*P. serrata* (Vell.) L.J.Gillespie	Davidse 10480 (MO)	Brazil	**MF502555**	**MF502480**	—	—	—	**MF502753**	**MF502820**
57	*P. serrata*	Forzza et al. 5328 (RB)	Brazil	**MF502556**	**MF502481**	**MF502622**	**MF502682**	—	**MF502754**	**MF502821**
58	*P. serrata*	Goldenberg et al. 1424 (RB)	Brazil	**MF502557**	**MF502482**	**MF502623**	**MF502683**	—	**MF502755**	**MF502822**
59	*P. serrata*	Peixoto et al. 4154 (MO)	Brazil	KP794458	**MF502483**	**MF502624**	—	—	**MF502756**	**MF502823**
60	*P. serrata*	Sartori & Pardo 11 (RB)	Brazil	**MF502558**	**MF502484**	**MF502625**	**MF502684**	—	**MF502757**	**MF502824**
61	*P. serrata*	Thomas 10221 (NY)	Brazil	**MF502559**	**MF502485**	**MF502626**	**MF502685**	—	—	**MF502825**
62	*P. stipellata* L.J.Gillespie	Aguilar 8193 (MO)	Costa Rica	KP794448	**MF502486**	**MF502627**	**MF502686**	**MF502876**	**MF502758**	**MF502826**
63	*P. stipellata*	Cardinal-McTeague 8 (CAN) [a]	Costa Rica	**MF502560**	**MF502487**	**MF502628**	**MF502687**	**MF502877**	**MF502759**	**MF502827**
64	*P. stipellata*	Ibarra Manriquez & Sinaca 1115 (MO)	Mexico	**MF502561**	**MF502488**	**MF502629**	**MF502688**	**MF502878**	—	**MF502828**
65	*P. stipellata*	Liesner 3088 (MO)	Costa Rica	KP794451	**MF502489**	**MF502630**	**MF502689**	**MF502879**	—	**MF502829**
66	*P. stipellata*	Morales & Rojas 5342 (MO)	Costa Rica	KP794450	**MF502490**	**MF502631**	**MF502690**	**MF502880**	**MF502760**	**MF502830**

67	*P. stipellata*	Refugio Cedillo Trigas 3510 (MO)	Mexico	**MF502562**	**MF502491**	**MF502632**	**MF502691**	**MF502881**	**MF502761**	**MF502831**
68	*P. stipellata*	Urbina 1155 (MO)	Nicaragua	KP794449	**MF502492**	**MF502633**	**MF502692**	**MF502882**	**MF502762**	**MF502832**
69	*P. supraglandulosa* L.J.Gillespie	Acevedo-Rodriguez 6022 (US)	Suriname	**MF502563**	**MF502493**	**MF502634**	**MF502693**	**MF502883**	**MF502763**	**MF502833**
70	*P. verrucosa* Sm.	Barrabe & Crozier 145 (US)	French Guiana	**MF502564**	**MF502494**	**MF502635**	**MF502694**	**MF502884**	**MF502764**	**MF502834**
71	*P. verrucosa*	Herrera & Kaemar 10073 (CAN)	Suriname	**MF502565**	**MF502495**	—	—	—	—	—
72	*P. verrucosa*	Hoffman 5917 (US)	Suriname	**MF502566**	**MF502496**	**MF502636**	**MF502695**	**MF502885**	**MF502765**	**MF502835**
73	*P. volubilis* L.	Bell 93-546 (US)	Peru	KP794447	**MF502497**	**MF502637**	**MF502696**	**MF502886**	**MF502766**	**MF502836**
74	*P. volubilis*	Burnham & Krings 1640 (MO)	Ecuador	KP794446	**MF502498**	**MF502638**	**MF502697**	**MF502887**	**MF502767**	**MF502837**
75	*P. volubilis*	Carrillo & Reyes 448 (MO)	Ecuador	**MF502567**	**MF502499**	**MF502639**	—	—	—	—
76	*P. volubilis*	Huamantupa 3500 (CAN)	Peru	**MF502568**	**MF502500**	**MF502640**	**MF502698**	—	—	—
77	*P. volubilis*	Jaramillo et al. 1237 (MO)	Bolivia	**MF502569**	**MF502501**	**MF502641**	**MF502699**	—	**MF502768**	**MF502838**
78	*P. volubilis*	Nee 55162 (MO)	Bolivia	KP794445	**MF502502**	**MF502642**	**MF502700**	**MF502888**	**MF502769**	**MF502839**
79	*P. volubilis*	Parada et al. 206 (CAN)	Bolivia	KP794444	**MF502503**	**MF502643**	**MF502701**	**MF502889**	**MF502770**	**MF502840**
80	*P. volubilis*	Teran et al. 2502 (MO)	Bolivia	**MF502570**	**MF502504**	**MF502644**	**MF502702**	—	**MF502771**	**MF502841**
81	*P. volubilis*	Valenzuela 8531 (MO)	Peru	**MF502571**	**MF502505**	—	—	—	—	—
82	*P. volubilis*	Wurdack s.n. (US) [a]	Smithsonian Greenhouse ex Peru	**MF502572**	**MF502506**	**MF502645**	**MF502703**	**MF502890**	**MF502772**	**MF502842**
83	*Romanoa tamnoides* (A.Juss.) Radcl.-Sm.	Fuentes 1848 (MO)	Bolivia	**MF502573**	**MF502507**	—	—	—	—	—
84	*R. tamnoides*	Raes & Terceros 177 (MO)	Bolivia	KP794435	**MF502508**	**MF502646**	**MF502704**	—	**MF502773**	**MF502843**
85	*R. tamnoides*	Raes et al. 211 (MO)	Bolivia	**MF502574**	**MF502509**	**MF502647**	**MF502705**	—	**MF502774**	**MF502844**
86	*R. tamnoides*	Zardini & Chaparro 50824 (MO)	Paraguay	**MF502575**	**MF502510**	**MF502648**	—	—	**MF502775**	**MF502845**

[a] = silica gel preserved voucher; all others from herbarium sheets

Table S2. Accession table of genomes/transcriptomes used in data-mining analyses.

No.	Taxon	Type	Assembly file	Reference
1	*Croton tiglium* L.	Transcriptome	VVPY_Croton_tiglium_-SOAPdenovo-Trans-assembly.fa	1KP Project
2	*Euphorbia mesembryanthemifolia* Jacq.	Transcriptome	LSLA_Euphorbia_mesembryanthemifolia_juv_-SOAPdenovo-Trans-assembly.fa	1KP Project
3	*E. mesembryanthemifolia*	Transcriptome	VPDX_Euphorbia_mesembryanthemifolia_mat_-SOAPdenovo-Trans-assembly.fa	1KP Project
4	*Jatropha curcas* L.	Draft genome	GCF_000696525.1_JatCur_1.0_rna.fna	Sato et al. 2011
5	*Manihot grahamii* Hook.	Transcriptome	XNLP_Manihot_grahamii_-SOAPdenovo-Trans-assembly.fa	1KP Project
6	*Plukenetia volubilis* L.	Transcriptome	PlutentiaAssemGADC01000001-GADC01066310.fasta	Wang et al. 2012
7	*Ricinus communis* L.	Draft genome	TIGR_castorWGS_release_0.1.cds.fsa	Chan et al. 2010
8	*R. communis*	Draft genome	TIGR_castorWGS_release_0.1.gene.fsa	Chan et al. 2010
9	*R. communis*	Transcriptome	PAZJ_Ricinus_communis_1kp_-SOAPdenovo-Trans-assembly.fa	1KP Project

Table S3. List of molecular markers, primer sequence, and amplification/sequencing protocols.

Name	Primer (5' to 3')	Reference	Recommended Protocol
Nuclear DNA (nDNA)			
External Transcribed Spacer (ETS)			
ETS F1	GTT ATG TTT GGY GTT TCG GSA ATG CT	Designed here	Amplification and sequencing: ETS Pluk F2 + 18S 5' R
ETS F2	ATG ATC GTT TGS TTT GGC AGG CTC	Designed here	Annealing temperature: 52ºC
18S 5' R	GAA TTA GTT CAT ACT TAC ACA TGC ATG	Designed here	
Internal Transcribed Spacer (ITS)			
ITS 5a [F]	CCT TAT CAT TTA GAG GAA GGA G	Stanford et al. 2000	Amplification and sequencing were done for the Full region (ITS 5a + u4) or in two overlapping fragments: ITS1 (ITS 5a + u2) and ITS2 (ITS p3 + u4). Sometimes ITS p2 and 4 were used as slightly internal sequencing primers. Annealing temperature: 55ºC
ITS u2 [R]	GCG TTC AAA GAY TCG ATG RTT C	Cheng et al. 2016	
ITS p2 [R]	GCC RAG ATA TCC GTT GCC GAG	Cheng et al. 2016	
ITS p3 [F]	YGA CTC TCG GCA ACG GAT A	Cheng et al. 2016	
ITS u4 [R]	RGT TTC TTT TCC TCC GCT TA	Cheng et al. 2016	
ITS 4 [R]	TCC TCC GCT TAT TGA TAT GC	White et al. 1990	
KEA1 intron 11			Amplification and sequencing: KEA1-i11 F + R Annealing temperature: 55ºC
KEA1-i11 F	GAA TCA GAT ATT GCK CCA TAT CGT G	Designed here	
KEA1-i11 R	AGA AGC TTT GGA TCA ATA GAC ATT C	Designed here	
KEA1 intron 17			Amplification and sequencing: KEA1-i17 F + R Annealing temperature: 65ºC
KEA1-i17 F	AGG TCC TTC ATA AAG TTG GTG CWG A	Designed here	
KEA1-i17 R	GTG CAA GAA CAG CAG CTG CCA ACT G	Designed here	
TEB exon 17			
TEB F	GCR AAG AAR TCA AGA ATG GTG CWC G	Designed here	Amplification and sequencing: TEB F + R2
TEB R1	CTC TCY TCA TCA GGC CAM GAA TCC A	Designed here	Annealing temperature: 55ºC
TEB R2	ATT SAT AGG ACC CTT CTC ACA GGC A	Designed here	
Plastid DNA (cpDNA)			
matK (full coding sequence)			
matK F1	AAT CCT CTA TCC TTG CTT CAA	Designed here	Amplification and sequencing were designed to capture the full coding region in three overlapping fragments, an effective protocol for poor quality DNA. matK Pluk F1 + R1, F2 + R2, and F3 + R3. Annealing temperature: 55ºC
matK R1	CTC ATG AAG AAA GAG TYG TAA TAA A	Designed here	
matK F2	AAT ACC TTA CCC CAT CCA TCT A	Designed here	
matK R2	ATT GGA ACT ATT GTA TCG AGT TTC	Designed here	
matK F3	GTA CGG AGT CAA ATG YTA GAA	Designed here	
matK R3	CCT TAC TCG TAT ACT GTA TGA GC	Designed here	
ndhF 3' (partial coding sequence)			Amplification and sequencing: ndhF 1318F + 2110Ri (or 1703R, to obtain a partial sequence for poorer quality samples) Annealing temperature: 49ºC
ndhF 1318F	GGA TTA ACY GCA TTT TAT ATG TTT CG	Olmstead and Sweere 1994	
ndhF 1703R	GGC TCC AAT AAA YAA AGT	Beilstein et al. 2006	
ndhF 2110Ri	TCA ATT ATT CGT TTA TCA A	Steinmann and Porter 2002	

All PCR amplifications used BIOLASE DNA Polymerase (Bioline) in 15uL reactions. Recipes included: 9.2uL H_2O; 1.5uL 10x NH_4 Reaction Buffer; 0.6uL 50mM $MgCl_2$ Solution [2.0mM]; 0.3uL 10mM dNTP Mix [0.2mM]; 0.375uL 10uM Primer (each) [0.25uM]; 1.5uL 1mg/mL Bovine Serum Albumin (BSA; Sigma Aldrich) [0.1 mg/mL]; 0.15uL 5U/uL BIOLASE DNA Polymerase [0.05U/uL]; 1uL Genomic DNA. Nuclear markers included 3uL 5M Betaine (Sigma Aldrich) [1M], in which case H_2O was reduced to 6.2uL. Thermocycler protocols followed: 3min 94ºC Initiation; 35 cycles of (i) 30s 94ºC Denaturing, (ii) 1m (see table) Annealing, (iii) 30s 72ºC Extension; 10min 72ºC Final extension.

Table S4. Biogeographical distribution matrix (A = Mexico to NW South America, B = Amazon/Guiana, C = Atlantic Forest, D = Tropical Africa, E = Madagascar, F = SE Asia).

Taxon	A	B	C	D	E	F
Haematostemon guianensis	0	1	0	0	0	0
Plukenetia africana	0	0	0	1	0	0
Plukenetia ankaranensis	0	0	0	0	1	0
Plukenetia brachybotrya	0	1	0	0	0	0
Plukenetia aff. *brachybotrya*	0	1	0	0	0	0
Plukenetia carabiasiae	1	0	0	0	0	0
Plukenetia cf. *carolis-vegae*	0	1	0	0	0	0
Plukenetia conophora	0	0	0	1	0	0
Plukenetia corniculata	0	0	0	0	0	1
Plukenetia decidua	0	0	0	0	1	0
Plukenetia lehmanniana	1	0	0	0	0	0
Plukenetia loretensis	0	1	0	0	0	0
Plukenetia madagascariensis	0	0	0	0	1	0
Plukenetia penninervia	1	0	0	0	0	0
Plukenetia cf. *penninervia*	1	0	0	0	0	0
Plukenetia polyadenia	0	1	0	0	0	0
Plukenetia serrata	0	0	1	0	0	0
Plukenetia stipellata	1	0	0	0	0	0
Plukenetia supraglandulosa	0	1	0	0	0	0
Plukenetia verrucosa	0	1	0	0	0	0
Plukenetia volubilis	0	1	0	0	0	0
Romanoa tamnoides	0	0	1	0	0	0

Table S5. Manual dispersal matrix between biogeographical areas (adjacent = 1.0, short-distance dispersal = 0.5, long-distance dispersal = 0.1).

0 to 35 Mya	Mex. to NW South Am. A	Amazon/ Guiana B	Atlantic Forest C	Trop. Africa D	Madagascar E	SE Asia F
Mex. to NW South Am.	1	-	-	-	-	-
Amazon/ Guiana	1.0	1	-	-	-	-
Atlantic Forest	0.1	0.5	1	-	-	-
Trop. Africa	0.1	0.1	0.1	1	-	-
Madagascar	0.1	0.1	0.1	0.5	1	-
SE Asia	0.1	0.1	0.1	0.1	0.1	1

Table S6. Trait matrix used in phylogenetic regression analysis with seed size. Plants size (0 = slender stems; 1 = robust to thick stems), seedling ecology (0 = light gap or forest edge; 1 = shade avoidance, canopy liana strategy), fruit type/ hypothesized dispersal mechanism (0 = dry, dehiscent/ scatter hoarding rodents; 1 = fleshy, indehiscent/ mammal predation), biome (0 = wet dominant; 1 = dry component), fire tolerance (0 = non-fire adapted; 1 = fire adapted).

Taxon	Plant size	Seedling ecology	Fruit type/ dispersal	Biome	Fire tolerance
Haematostemon guianensis	1	0	0	0	0
Romanoa tamnoides	0	0	0	0	0
Plukenetia africana	0	0	0	1	1
Plukenetia ankaranensis	1	0	0	1	0
Plukenetia brachybotrya	0	0	0	0	0
Plukenetia aff. *brachybotrya*	0	0	0	0	0
Plukenetia carabiasiae	1	1	0	0	0
Plukenetia cf. *carolis-vegae*	1	0	0	0	0
Plukenetia conophora	1	1	1	0	0
Plukenetia corniculata	0	0	0	0	0
Plukenetia decidua	1	0	0	1	0
Plukenetia huayllabambana	1	0	0	0	0
Plukenetia lehmanniana	1	0	1	0	0
Plukenetia loretensis	0	0	0	0	0
Plukenetia penninervia	0	0	0	0	0
Plukenetia cf. *penninervia*	0	0	0	0	0
Plukenetia polyadenia	1	1	1	0	0
Plukenetia serrata	1	0	1	0	0
Plukenetia stipellata	0	0	0	0	0
Plukenetia supraglandulosa	0	0	0	0	0
Plukenetia verrucosa	0	0	0	0	0
Plukenetia volubilis	0	0	0	0	0

Table S7. The best evolutionary model for seed size based on ΔAIC and Akaike weights (w_i), using the modified *Plukenetia* BEAST chronogram, $\log_{10}$(estimated seed volume), and the fitContinuous function in GEIGER.

Model	AIC_c	ΔAIC	w_i
Brownian motion	50.03442	0.0	0.6584417
Ornstein-Uhlenbeck	52.73257	2.69815	0.1708530
Early Burst	52.73430	2.69988	0.1707053
