# Supplementary material for: Seed size evolution and biogeography of Plukenetia (Euphorbiaceae), a pantropical genus with traditionally cultivated oilseed species
Source: BMC Evol Biol. 2019 Jan 22;19:29. doi: 10.1186/s12862-018-1308-9 (PMC6341577; doi:10.1186/s12862-018-1308-9)

Fig. S1 Primer map for new (*KEA1* introns 11 and 17, *TEB* exon 17) and redesigned (ETS, *matK*) molecular markers.

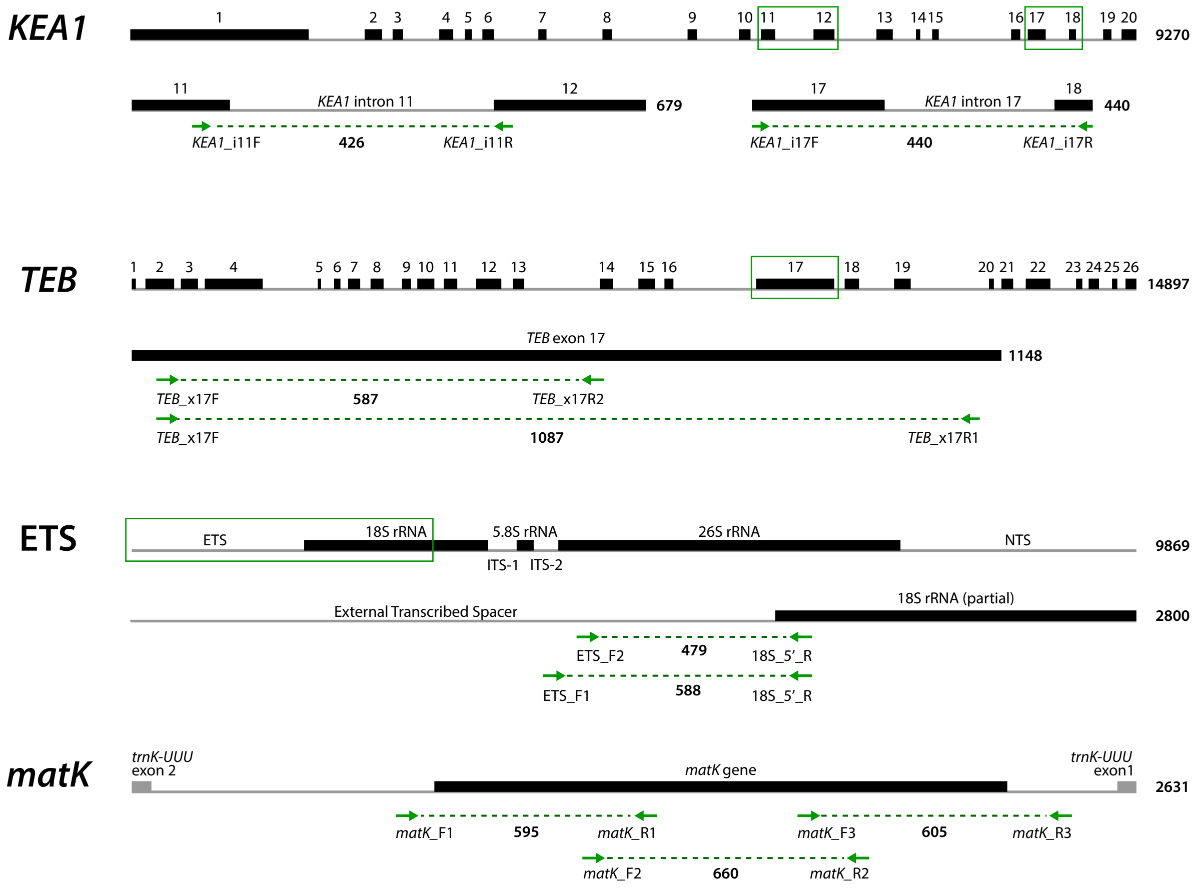

Fig. S2 a
nrDNA ETS
HKY+G
500 ML Bootstrap Replicates

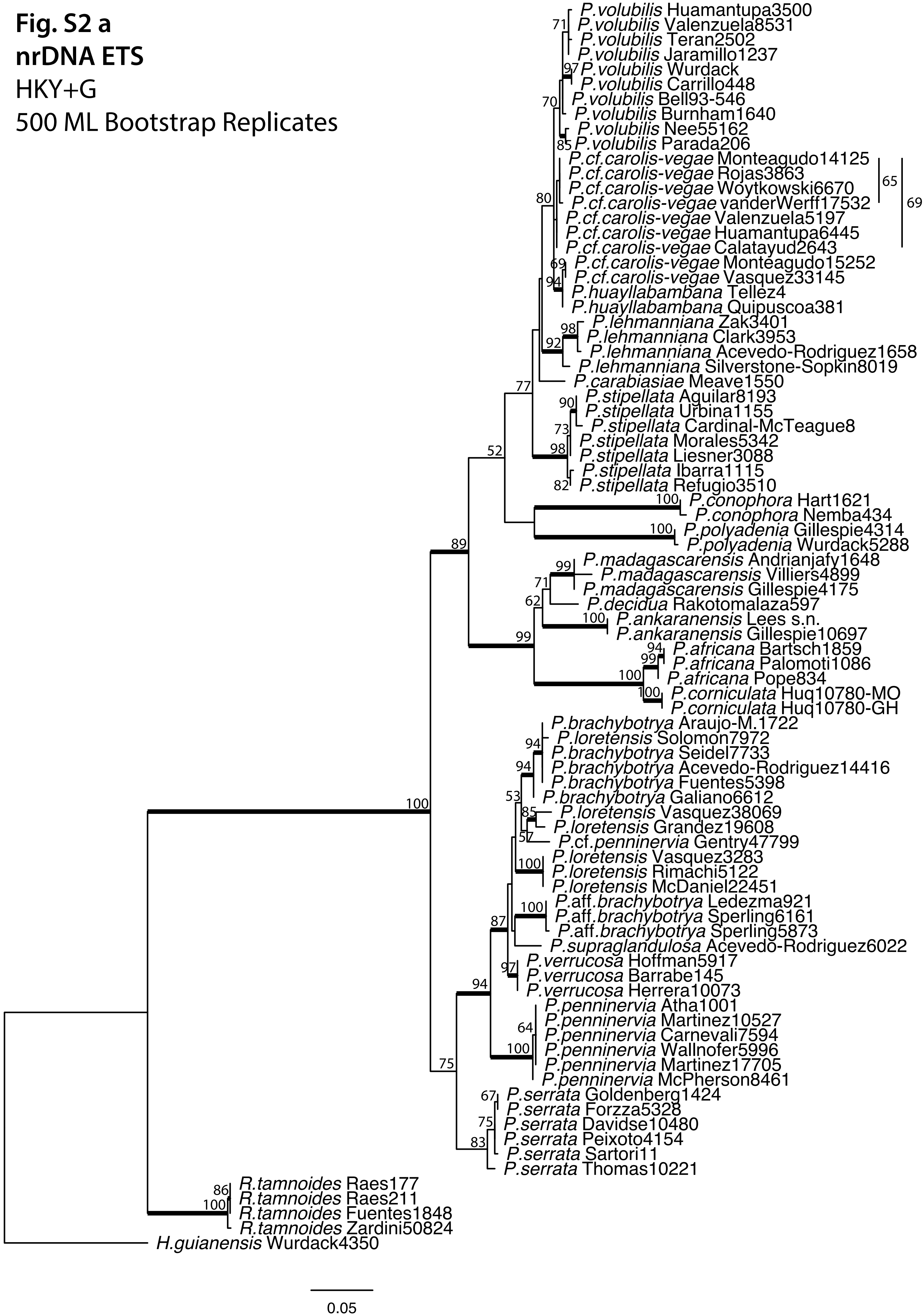

Fig. S2 b
nrDNA ITS
GTR+I+G
500 ML Bootstrap Replicates

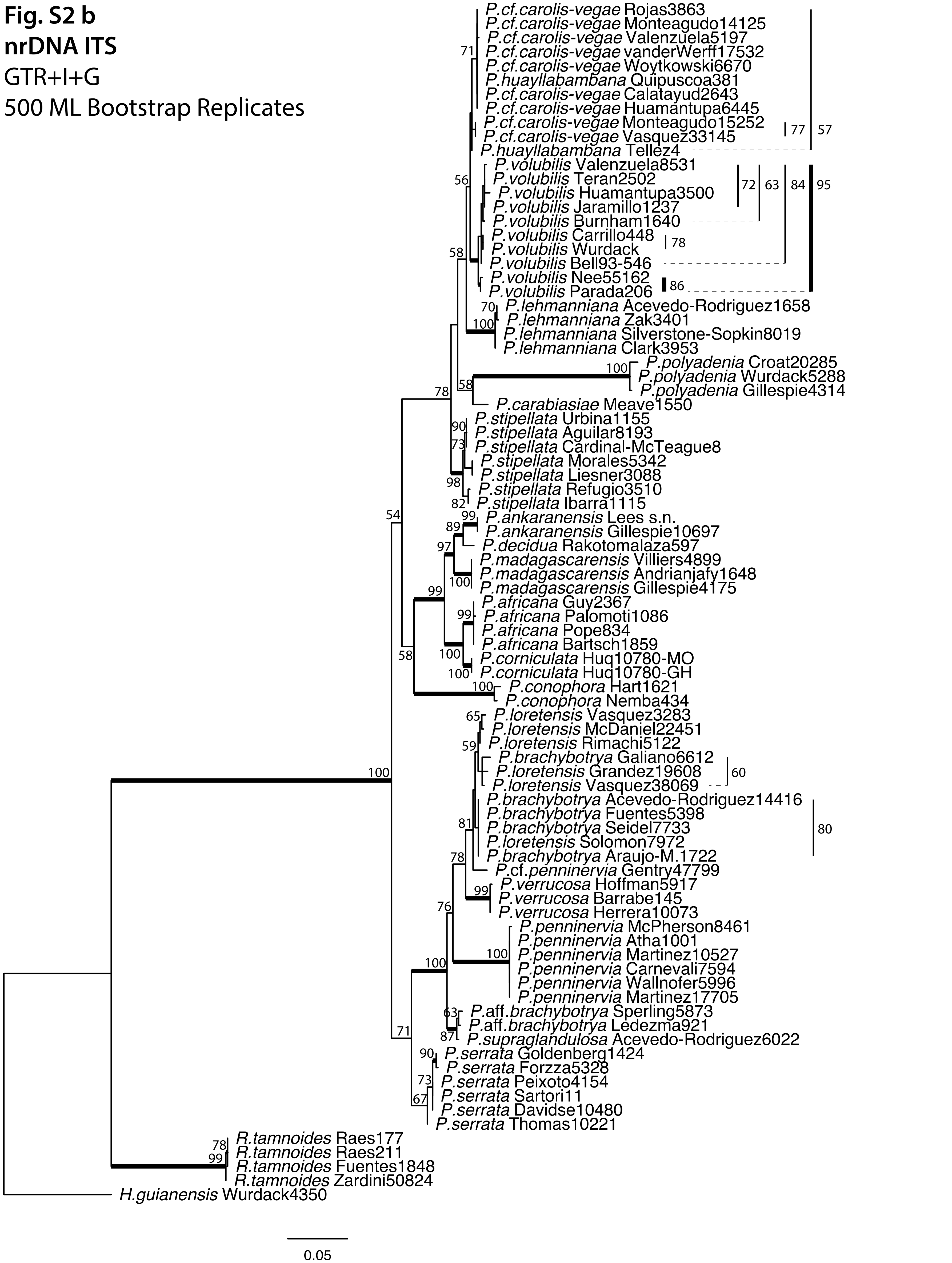

Fig. S2 c
nDNA *KEA1* intron 11
GTR+G
500 ML Bootstrap Replicates

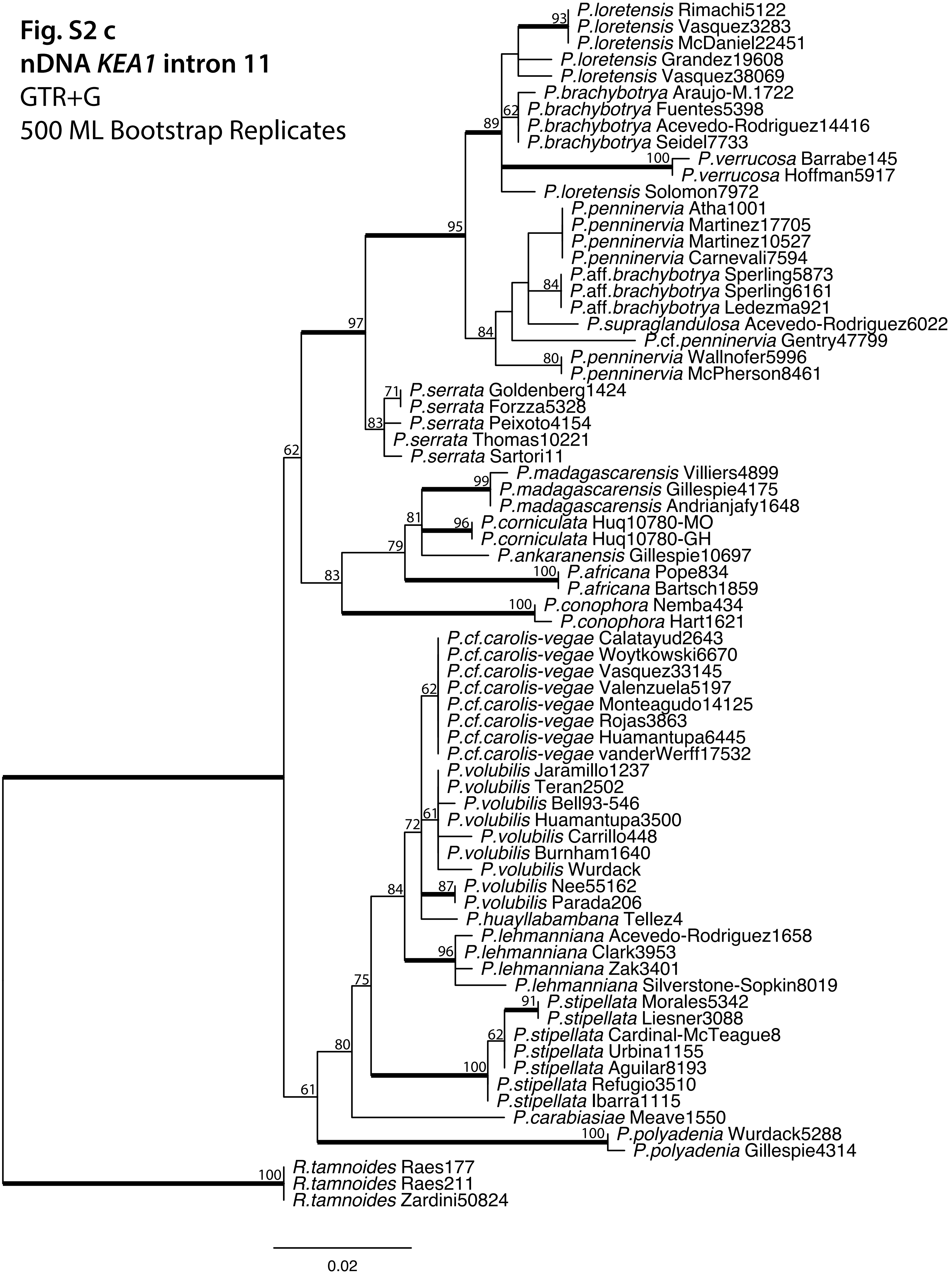

Fig. S2 d
nDNA *KEA1* intron 17
HKY+I
500 ML Bootstrap Replicates

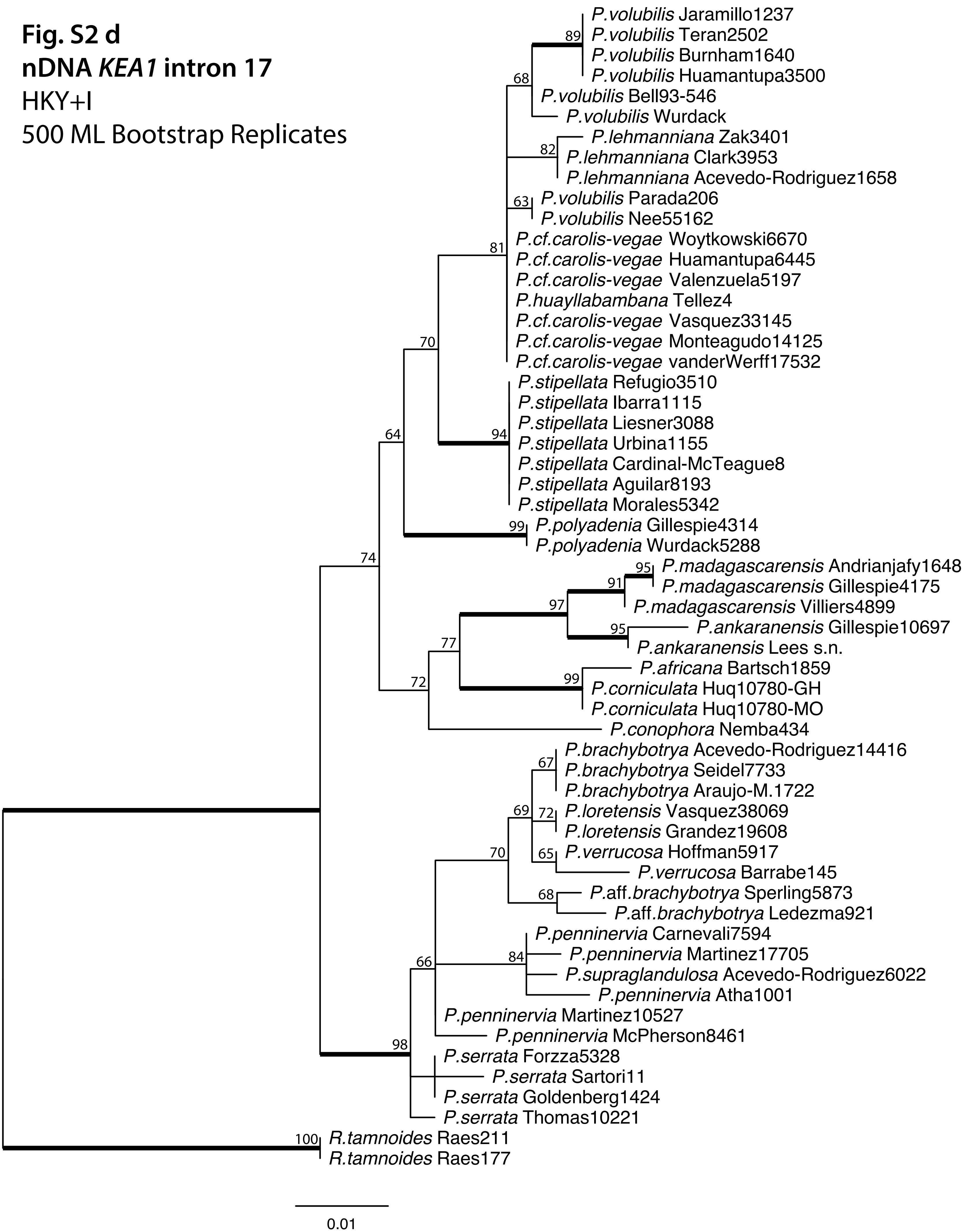

Fig. S2 e
nDNA *TEB* exon 17
HKY+I
500 ML Bootstrap Replicates

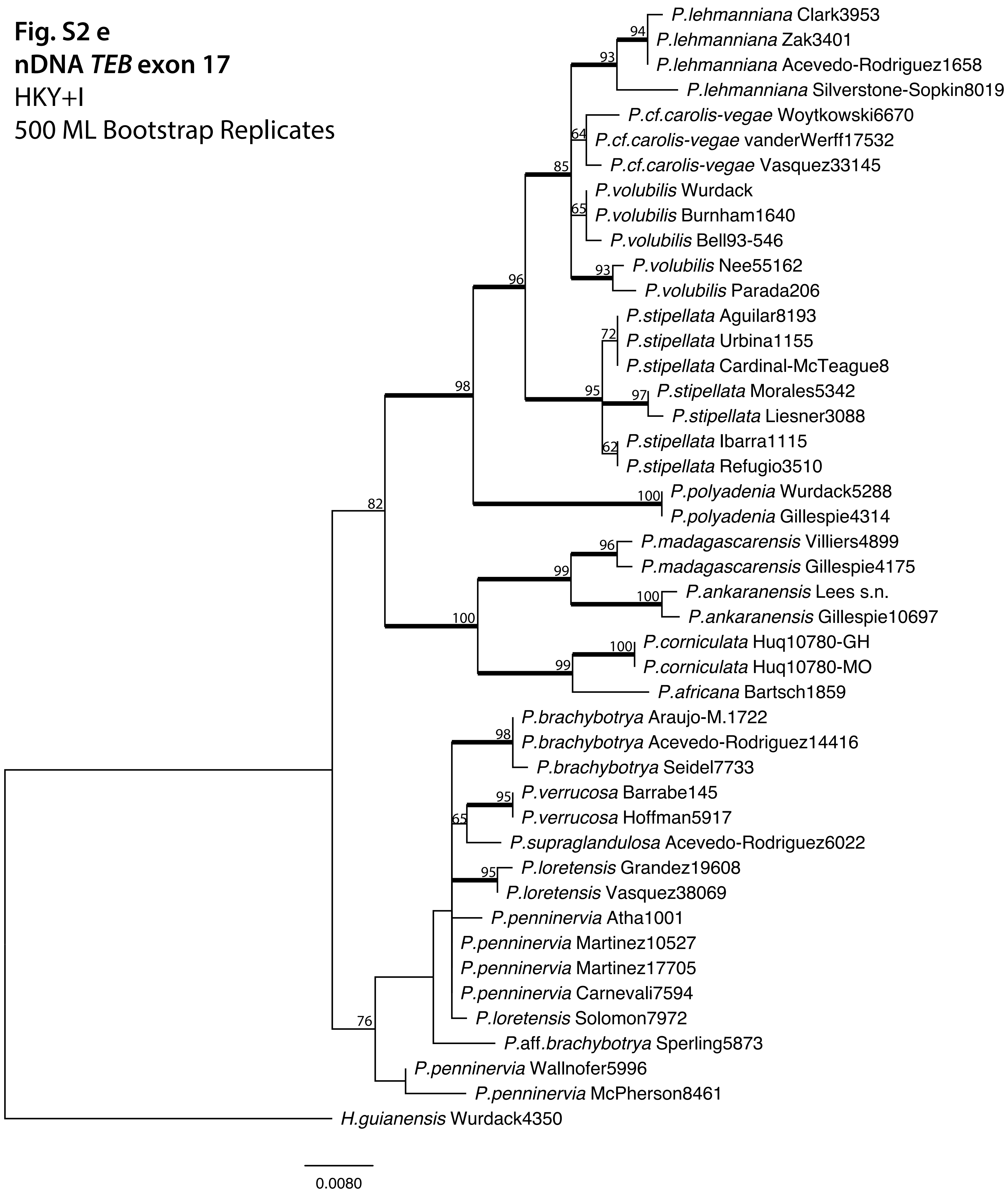

Fig. S2 f
cpDNA *matK*
GTR+G
500 ML Bootstrap Replicates

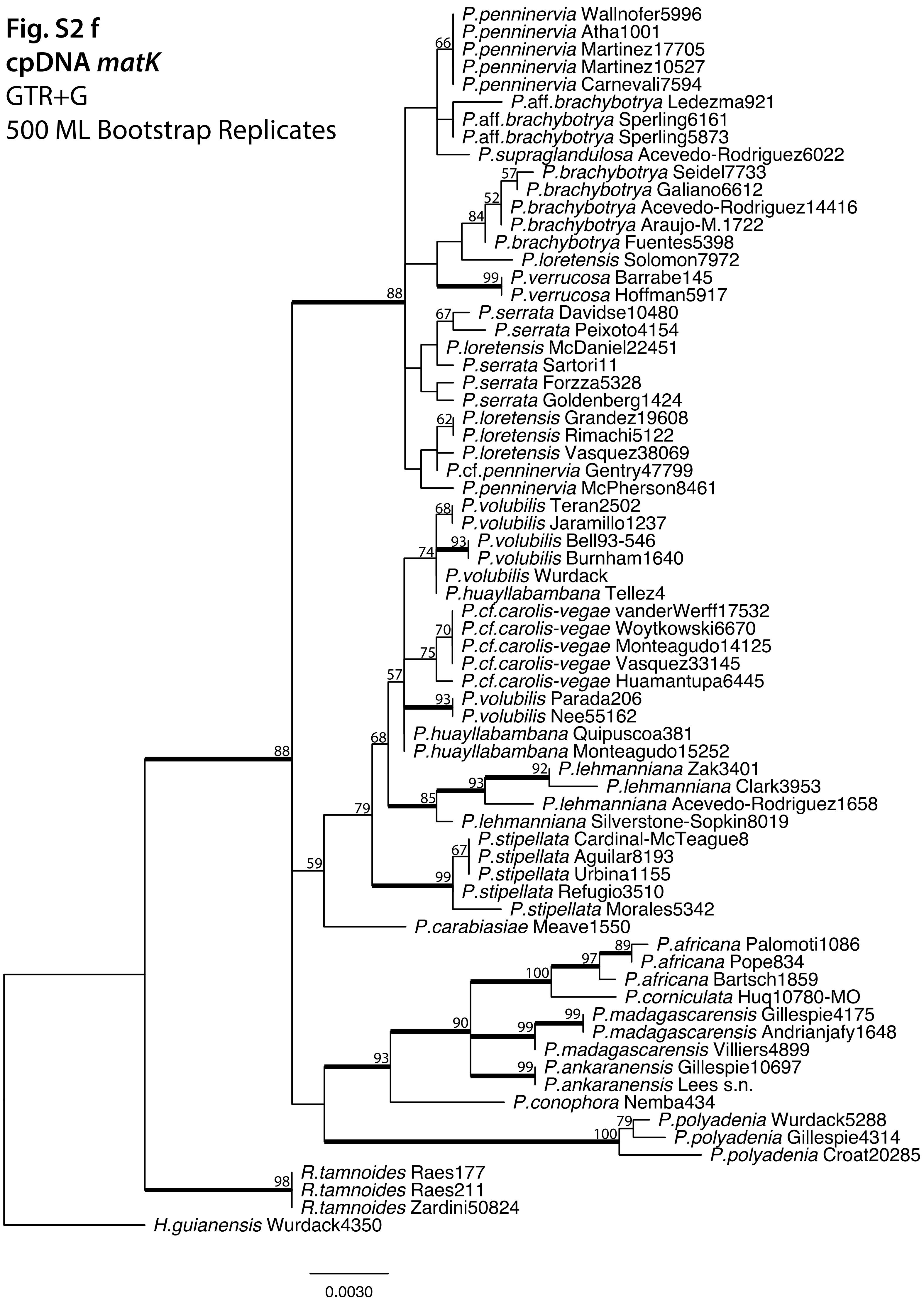

Fig. S2 g
cpDNA *ndhF*
GTR+I+G
500 ML Bootstrap Replicates

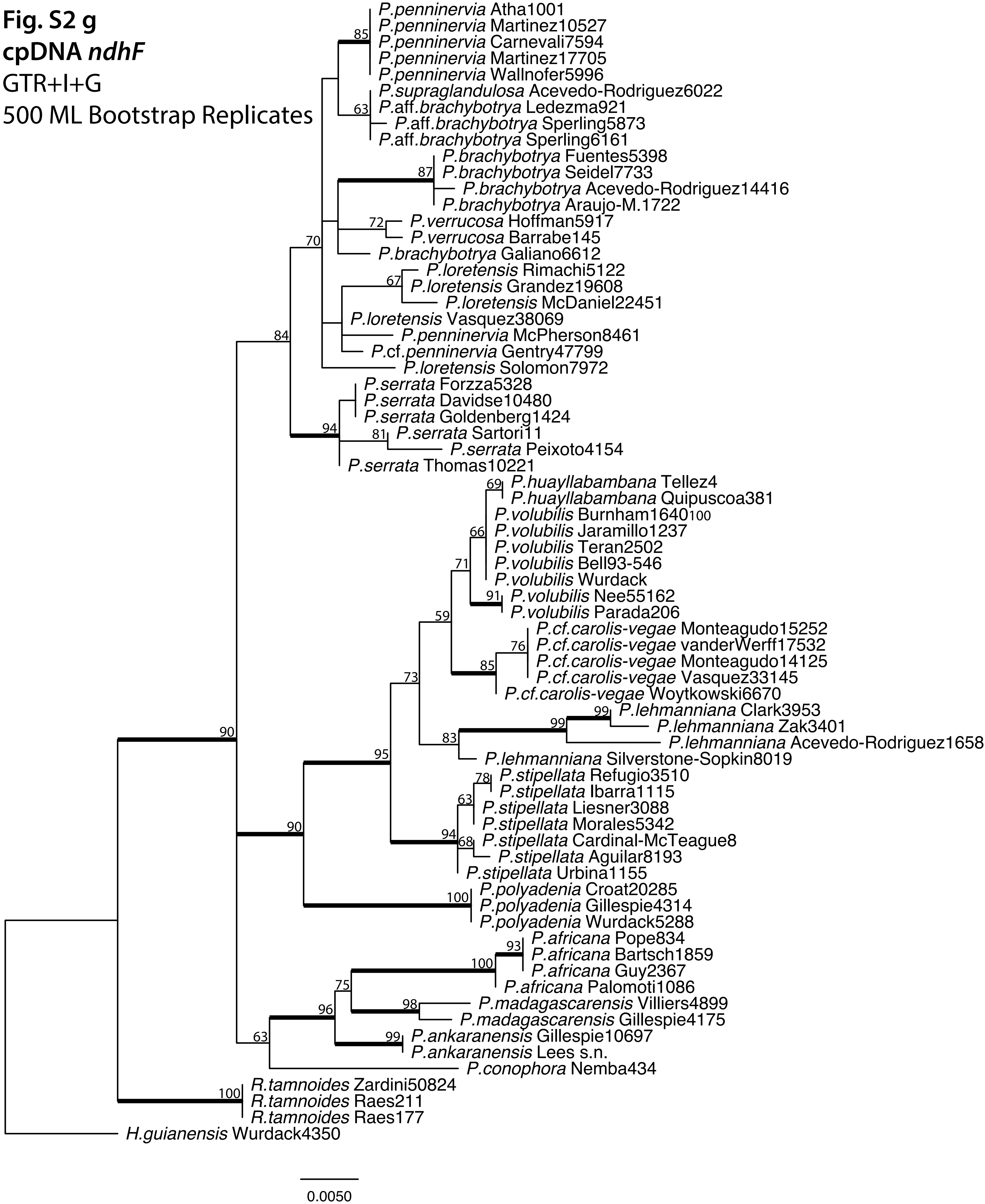

Fig. S3 Bayesian maximum clade credibility tree based on (a) plastid DNA (cpDNA) two marker, 74 accession dataset, and (b) nuclear DNA (nDNA) five marker, 86 accession dataset, for *Plukenetia* and Plukenetiinae outgroups. Maximum parsimony bootstrap percentage (MPBP) and Bayesian posterior probability (PP) support values > 50% are indicated on each branch. Branches in bold indicate strong support (≥ 85 MPBP and ≥ 0.95 PP). Grey boxes highlight strongly supported topological incongruences.

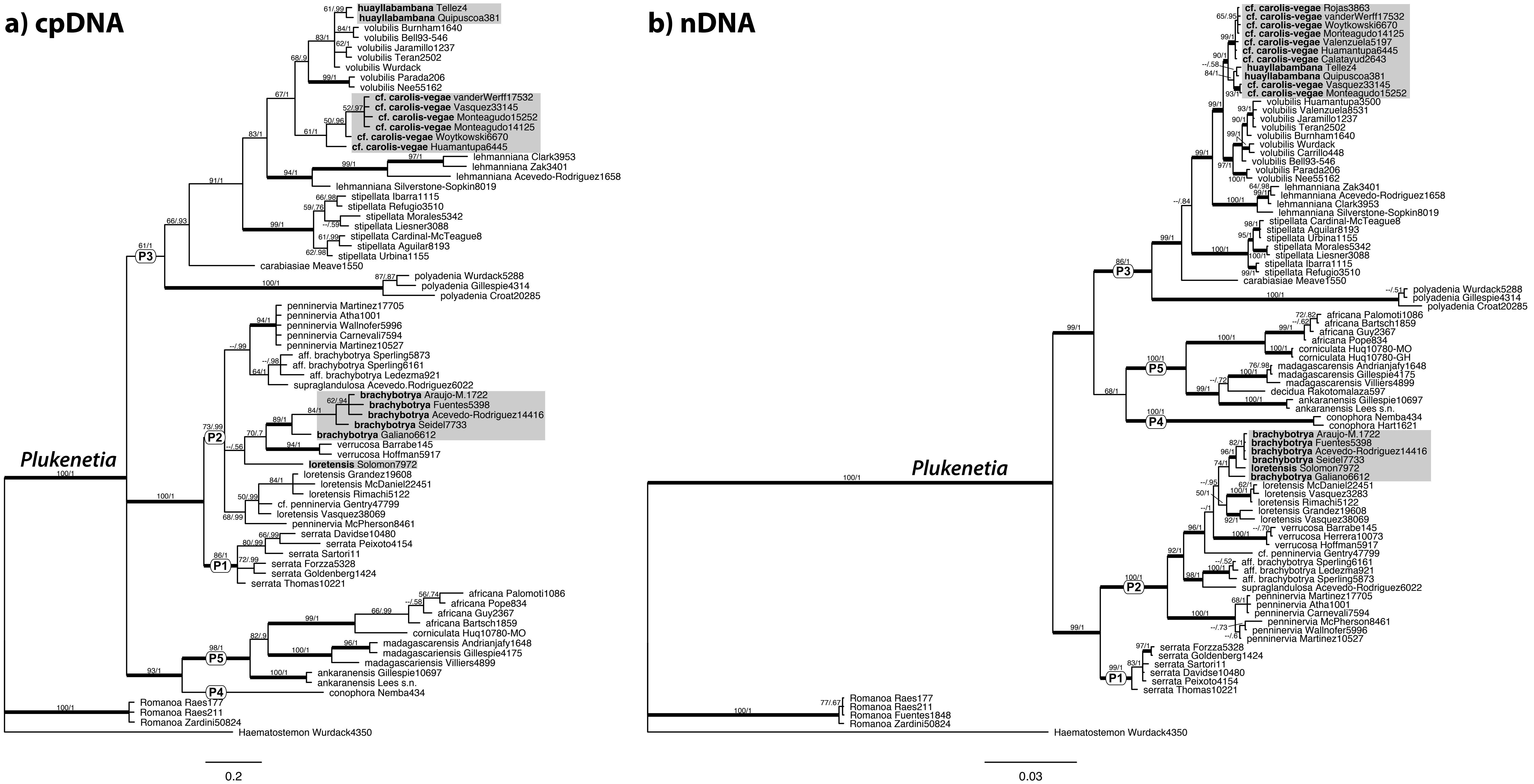

Fig. S4 BEAST chronogram of *Plukenetia* and Plukenetiinae outgroups inferred from the combined seven marker (cpDNA and nDNA), 83 accession dataset and two normal-distribution priors (indicated in red) based on previous subfamily Acalyphoideae estimates using three fossil calibrations. Numbers at each node indicate mean age estimates, and blue bars the 95% highest posterior density confidence interval.

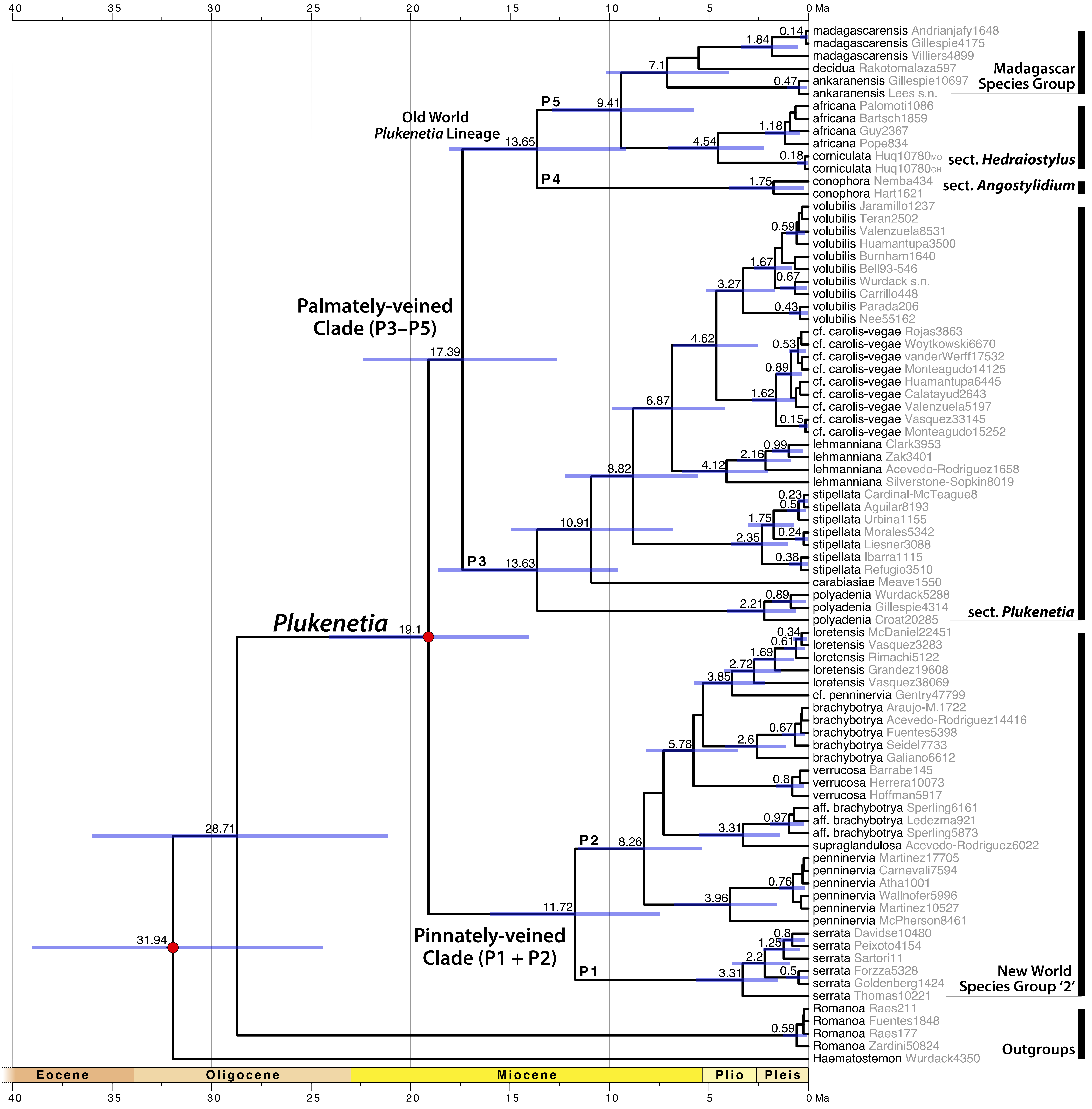

Fig. S5 a BioGeoBEARS DEC+J on *Plukenetia*

ancstates: global optim, 2 areas max. d=0; e=0; j=0.3997; LnL=−27.26

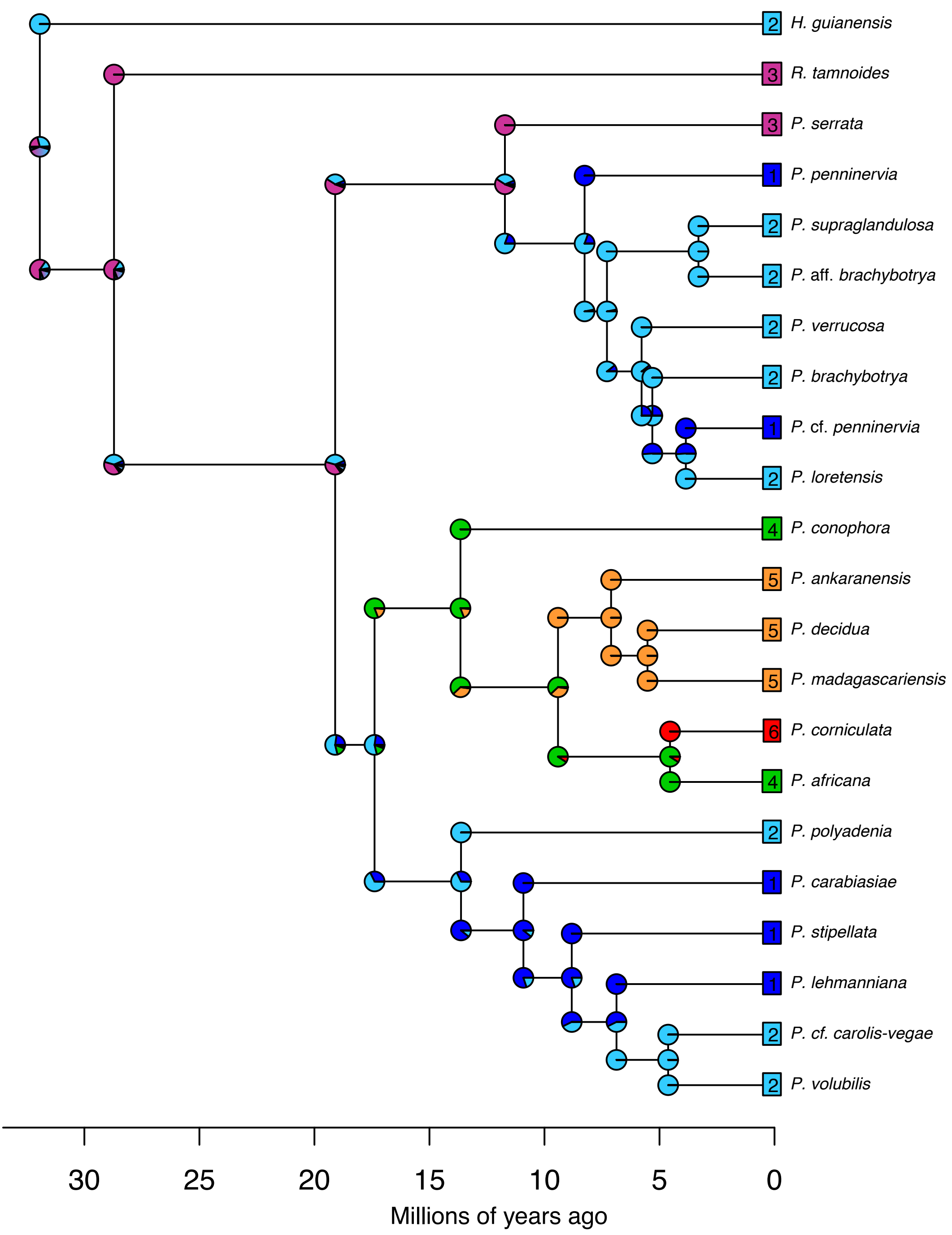

Fig. S5 b BioGeoBEARS DEC on *Plukenetia*

ancstates: global optim, 2 areas max. d=0.034; e=0.0163; j=0; LnL=−46.67

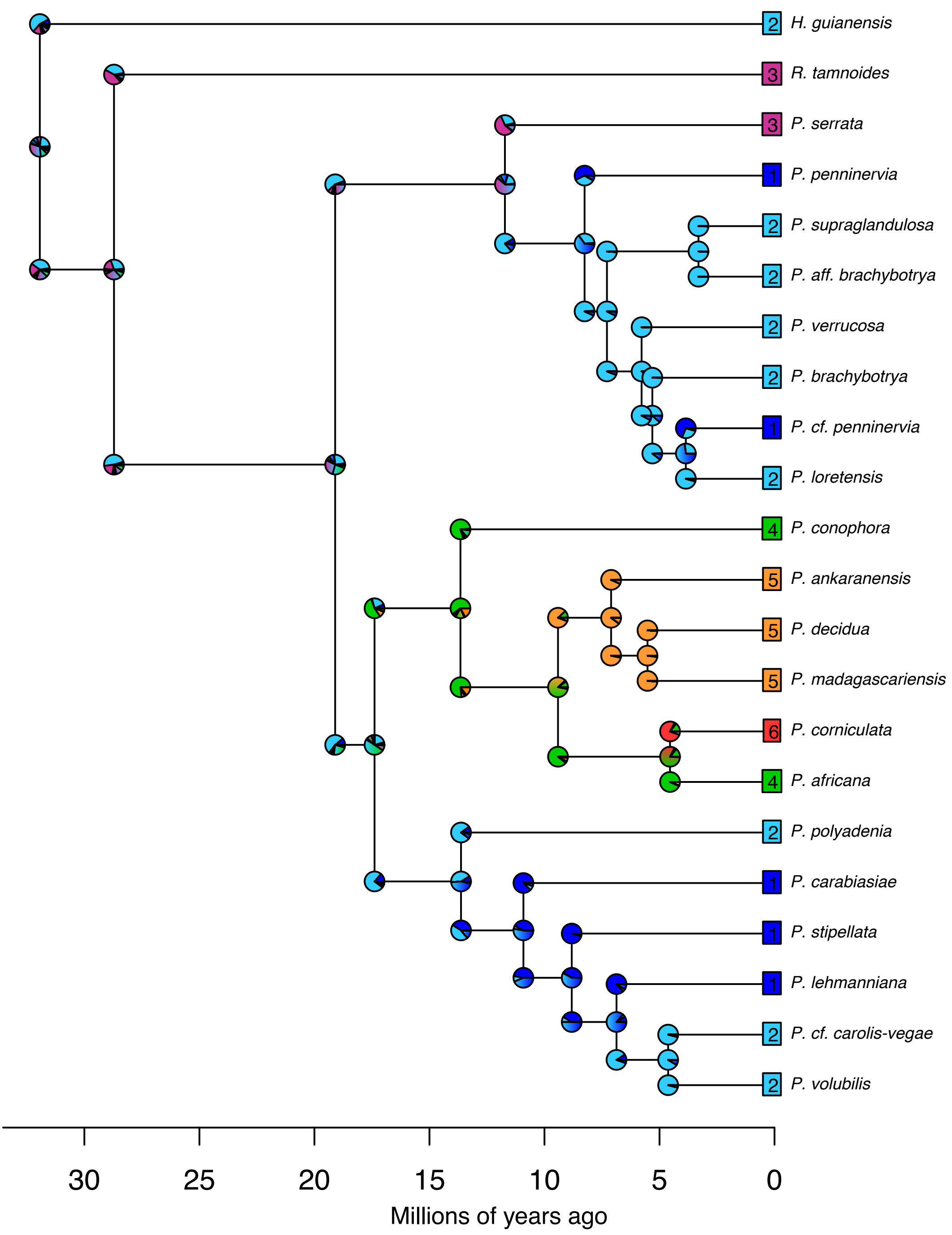

Fig. S6 UPGMA clustering analysis of log10 transformed seed dimensions (length, width, thickness) for 190 accessions of *Plukenetia*.

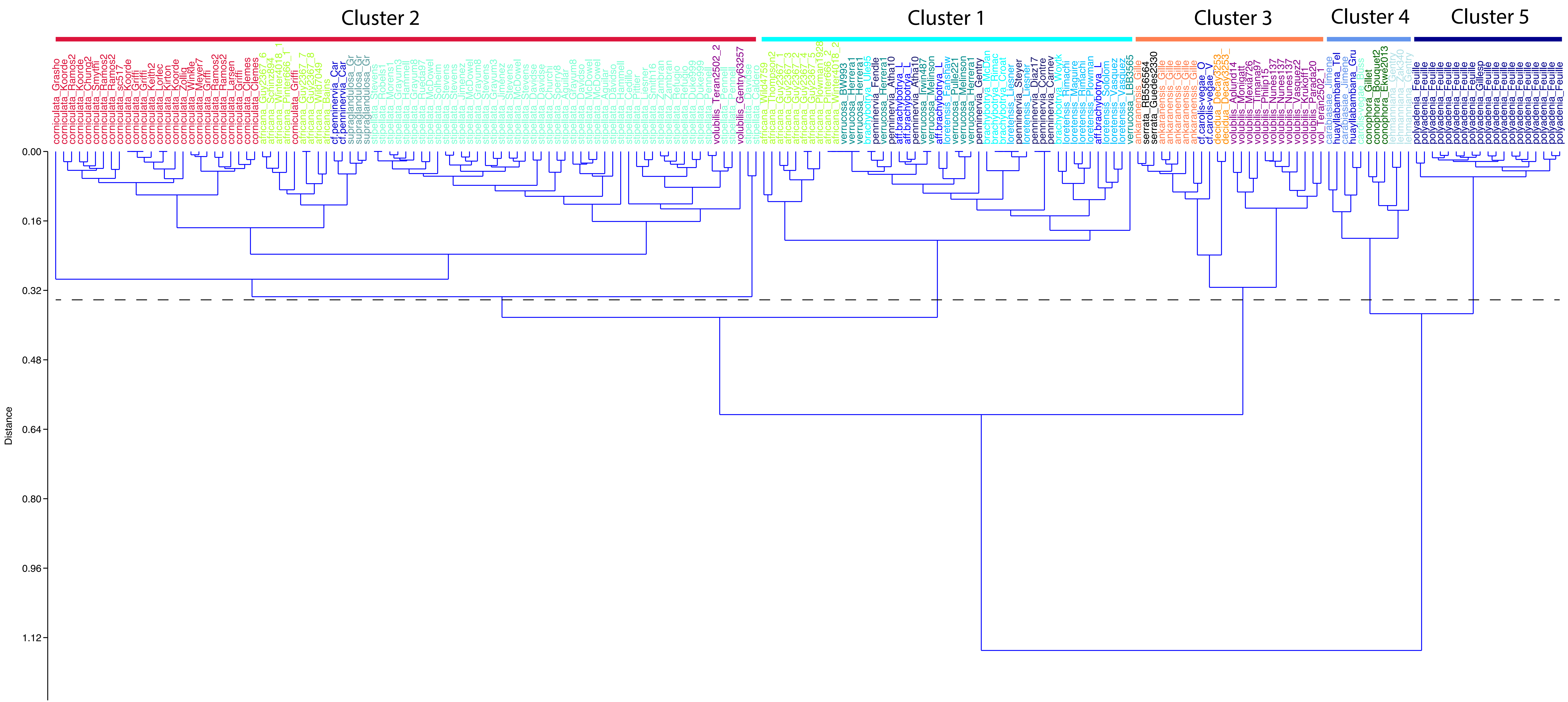

Fig. S7 Principal components analysis of log10 transformed seed dimensions (length, width, thickness) for 190 accessions of *Plukenetia*.

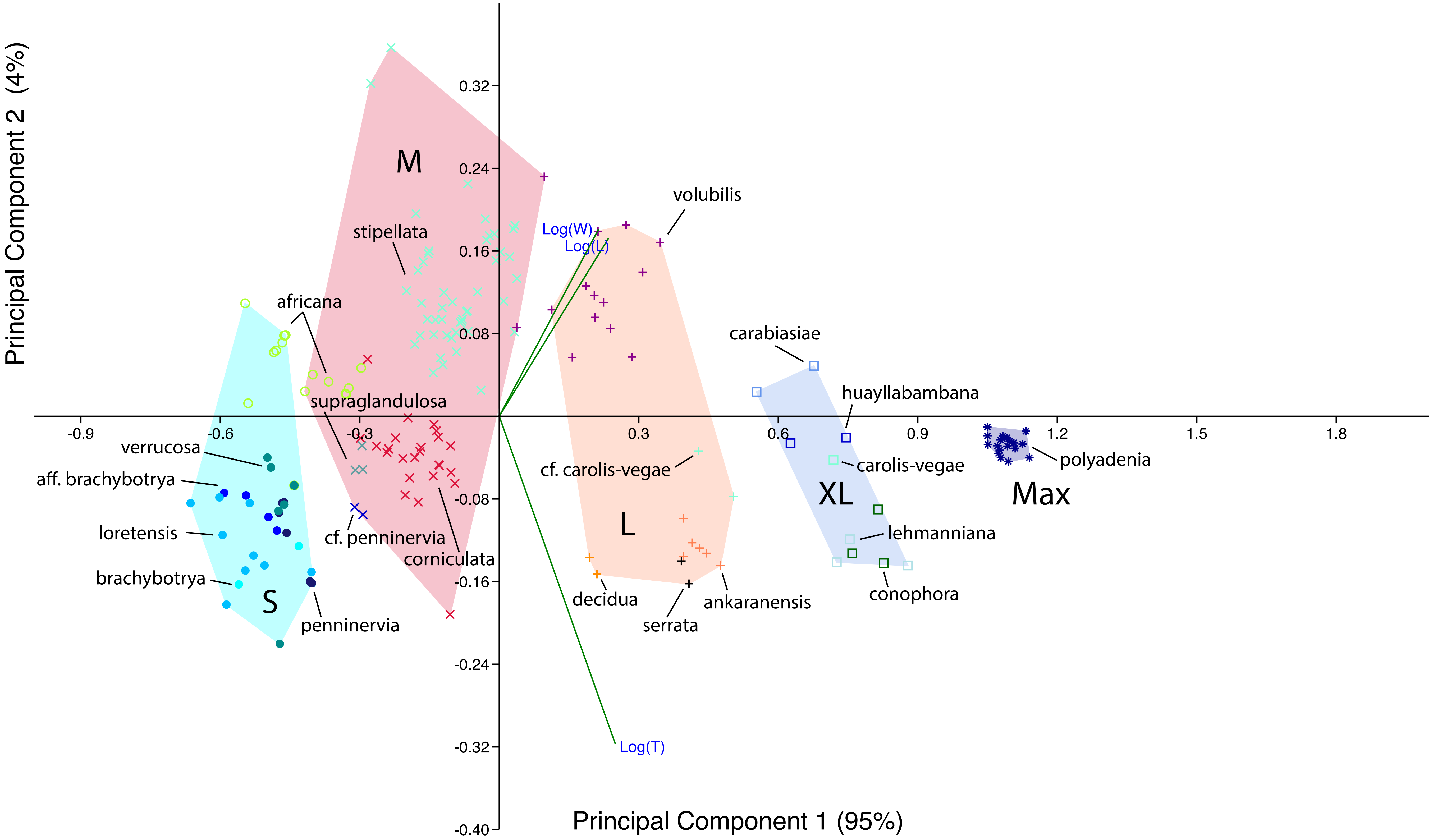

Fig. S8 Posterior distributions of the correlation coefficients (*r*) between the liabilities of seed size and (a) plant size, (b) fruit type, (c) seedling ecology, (d) fire tolerance, and (e) biome type, under the threshold model.

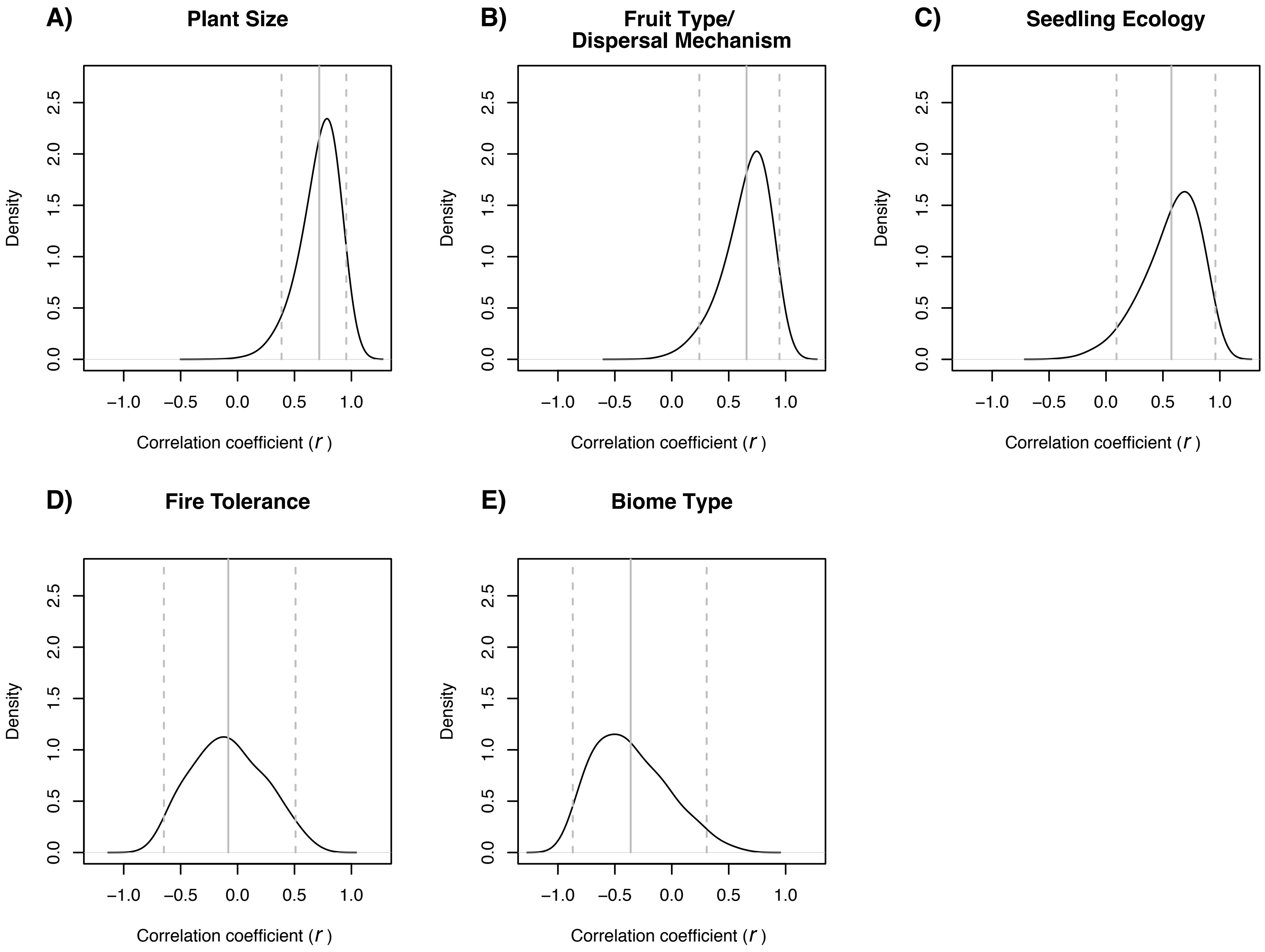

Supplement: Supplementary file 4 — Figure S1. Primer map for new (KEA1 introns 11 and 17, TEB exon 17) and redesigned (ETS, matK) molecular markers. Figure S2. Shortest maximum likelihood tree for incongruence analysis of individual markers: (a) ETS, (b) ITS, (c) KEA1 intron 11, (d) KEA1 intron 17, (e) TEB exon 17, (f) matK, and (g) ndhF. Bootstrap percentages based on 500 replicates. Well-supported branches (> 85% maximum likelihood bootstrap percentage; MLBP) are in bold. Figure S3. Bayesian maximum clade credibility tree based on (a) plastid DNA (cpDNA) two marker, 74 accession dataset, and (b) nuclear DNA (nDNA) five marker, 86 accession dataset, for Plukenetia and Plukenetiinae outgroups. Maximum parsimony bootstrap percentage (MPBP) and Bayesian posterior probability (PP) support values > 50% are indicated on each branch. Branches in bold indicate strong support (≥ 85 MPBP and ≥ 0.95 PP). Grey boxes highlight strongly supported topological incongruences. Figure S4. BEAST chronogram of Plukenetia and Plukenetiinae outgroups inferred from the combined seven marker (cpDNA and nDNA), 83 accession dataset and two normal-distribution priors (indicated in red) based on previous subfamily Acalyphoideae estimates using three fossil calibrations. Numbers at each node indicate mean age estimates, and blue bars the 95% highest posterior density confidence interval. Figure S5. BioGeoBEARS ancestral range estimation probabilities for each corner and node under (a) DEC + J and (b) DEC. Figure S6. UPGMA clustering analysis of log10 transformed seed dimensions (length, width, thickness) for 190 accessions of Plukenetia. Figure S7. Principal components analysis of log10 transformed seed dimensions (length, width, thickness) for 190 accessions of Plukenetia. Figure S8. Posterior distributions of the correlation coefficients (r) between the liabilities of seed size and (a) plant size, (b) fruit type, (c) seedling ecology, (d) fire tolerance, and (e) biome type, under the threshold model. (PDF 2520 kb) [file 12862_2018_1308_MOESM4_ESM.pdf]
